# The human milk proteome and allergy of mother and child: Exploring associations with protein abundances and protein network connectivity

**DOI:** 10.3389/fimmu.2022.977470

**Published:** 2022-10-13

**Authors:** Pieter M. Dekker, Meghan B. Azad, Sjef Boeren, Piushkumar J. Mandhane, Theo J. Moraes, Elinor Simons, Padmaja Subbarao, Stuart E. Turvey, Edoardo Saccenti, Kasper A. Hettinga

**Affiliations:** ^1^ Food Quality and Design Group, Wageningen University and Research, Wageningen, Netherlands; ^2^ Laboratory of Biochemistry, Wageningen University and Research, Wageningen, Netherlands; ^3^ Department of Pediatrics and Child Health, University of Manitoba, Winnipeg, MB, Canada; ^4^ Manitoba Interdisciplinary Lactation Centre (MILC), Children’s Hospital Research Institute of Manitoba, Winnipeg, MB, Canada; ^5^ Department of Pediatrics, University of Alberta, Edmonton, AB, Canada; ^6^ Division of Respiratory Medicine, Department of Pediatrics, Hospital for Sick Children, University of Toronto, Toronto, ON, Canada; ^7^ Department of Physiology, University of Toronto, Toronto, ON, Canada; ^8^ Department of Pediatrics, University of British Columbia, Vancouver, BC, Canada; ^9^ Laboratory of Systems and Synthetic Biology, Wageningen University and Research, Wageningen, Netherlands

**Keywords:** breastmilk, milk proteome, allergic disease, allergy development, immunology of human milk, differential network analysis, allergen, immunomodulatory

## Abstract

**Background:**

The human milk proteome comprises a vast number of proteins with immunomodulatory functions, but it is not clear how this relates to allergy of the mother or allergy development in the breastfed infant. This study aimed to explore the relation between the human milk proteome and allergy of both mother and child.

**Methods:**

Proteins were analyzed in milk samples from a subset of 300 mother-child dyads from the Canadian CHILD Cohort Study, selected based on maternal and child allergy phenotypes. For this selection, the definition of “allergy” included food allergy, eczema, allergic rhinitis, and asthma. Proteins were analyzed with non-targeted shotgun proteomics using filter-aided sample preparation (FASP) and nanoLC-Orbitrap-MS/MS. Protein abundances, based on label-free quantification, were compared using multiple statistical approaches, including univariate, multivariate, and network analyses.

**Results:**

Using univariate analysis, we observed a trend that milk for infants who develop an allergy by 3 years of age contains higher abundances of immunoglobulin chains, irrespective of the allergy status of the mother. This observation suggests a difference in the milk’s immunological potential, which might be related to the development of the infant’s immune system. Furthermore, network analysis showed overall increased connectivity of proteins in the milk of allergic mothers and milk for infants who ultimately develop an allergy. This difference in connectivity was especially noted for proteins involved in the protein translation machinery and may be due to the physiological status of the mother, which is reflected in the interconnectedness of proteins in her milk. In addition, it was shown that network analysis complements the other methods for data analysis by revealing complex associations between the milk proteome and mother-child allergy status.

**Conclusion:**

Together, these findings give new insights into how the human milk proteome, through differences in the abundance of individual proteins and protein-protein associations, relates to the allergy status of mother and child. In addition, these results inspire new research directions into the complex interplay of the mother-milk-infant triad and allergy.

## Introduction

Having an allergy can strongly impact someone’s quality of life in terms of dietary, social, and psychological factors. The burden of allergic diseases for healthcare is increasing in western countries (1). In an attempt to decrease these socioeconomic burdens, a primary concern is to determine in which time frame the development of allergic diseases is triggered (window of opportunity) and how this can be prevented. This time frame centers around the first years, as allergic diseases often manifest themselves in the first years of life, and healthy development of the infant’s immune system is crucial for later immune health (2).

The role of human milk in the development of allergic diseases has received considerable attention in recent years (3–5). Breastfed babies receive a spectrum of nutrients through human milk, in a stage of life that is crucial for the development of the immune system. Several components in human milk have functional properties that could play a role in immune development, such as antioxidant, antibacterial, and immunomodulating properties; e.g., vitamins, antibodies, and cytokines, respectively (6). The effect of breastfeeding on the development of allergic diseases is complex and has been the subject of several epidemiological studies in the last decades (7–9), although meta-analyses do not show conclusive evidence for an allergy preventing effect of breastfeeding (10, 11). For example, Kull et al. showed that exclusively breastfed (≥ 4 months) children in the general population had a reduced risk of allergic sensitization and asthma compared to children breastfed for less than 4 months (8), while Mihrshahi et al. (9) reported no significant association between onset of atopy and duration of exclusive breastfeeding. One explanation for these contradicting findings could be differences in the definition of the outcomes. However, it could also be due to the individual-specific composition of human milk which relates to, amongst other factors, maternal genetics or diet (12, 13).

It is possible that specific components in human milk with levels determined by individual-specific factors could influence the development of the immune system of the breastfed child. Proteins are a particularly important group of such human milk components with immunomodulatory potential, including immunoglobulins (Igs), cytokines, and dietary antigens.

Thus far, several studies have demonstrated the importance of human milk proteins for the development of the infant’s immune system (14–17). In a cohort study including 398 children, Munblit et al. (14) found that higher levels of TGF*β*2 in human milk were related to a higher occurrence of eczema in the infant. Österlund et al. (15) showed that eosinophil cationic protein (ECP), a marker of eosinophil degranulation, was present at higher levels in human milk consumed by children that developed cow’s milk allergy or atopic dermatitis. In another study, Järvinen et al. (16) reported that infants who received human milk with low levels of total immunoglobulin A (IgA) were more likely to develop cow’s milk allergy. A more recent study conducted by Michel et al. (17) showed interdependencies between maternal allergy status, risk of allergy development in the infant, and IgA, TGF*β*1, and TGF*β*2 levels in human milk.

In addition to these studies showing the importance of human milk proteins, some studies show the presence of dietary allergens in human milk and their possible relation with maternal and infant allergy status. It was shown, for example, that a bovine milk allergen is more abundant in milk from allergic mothers (18) and that the presence of such allergens in the milk might result in tolerance induction (19). In addition, it was shown by Adel-Patient et al. (20) that sensitized mice who were exposed to bovine *β*-lactoglobulin (BLG) during lactation transferred protection for this allergen to their offspring at a level that correlated with the level of BLG-specific antibodies in the milk.

**Figure 1:**
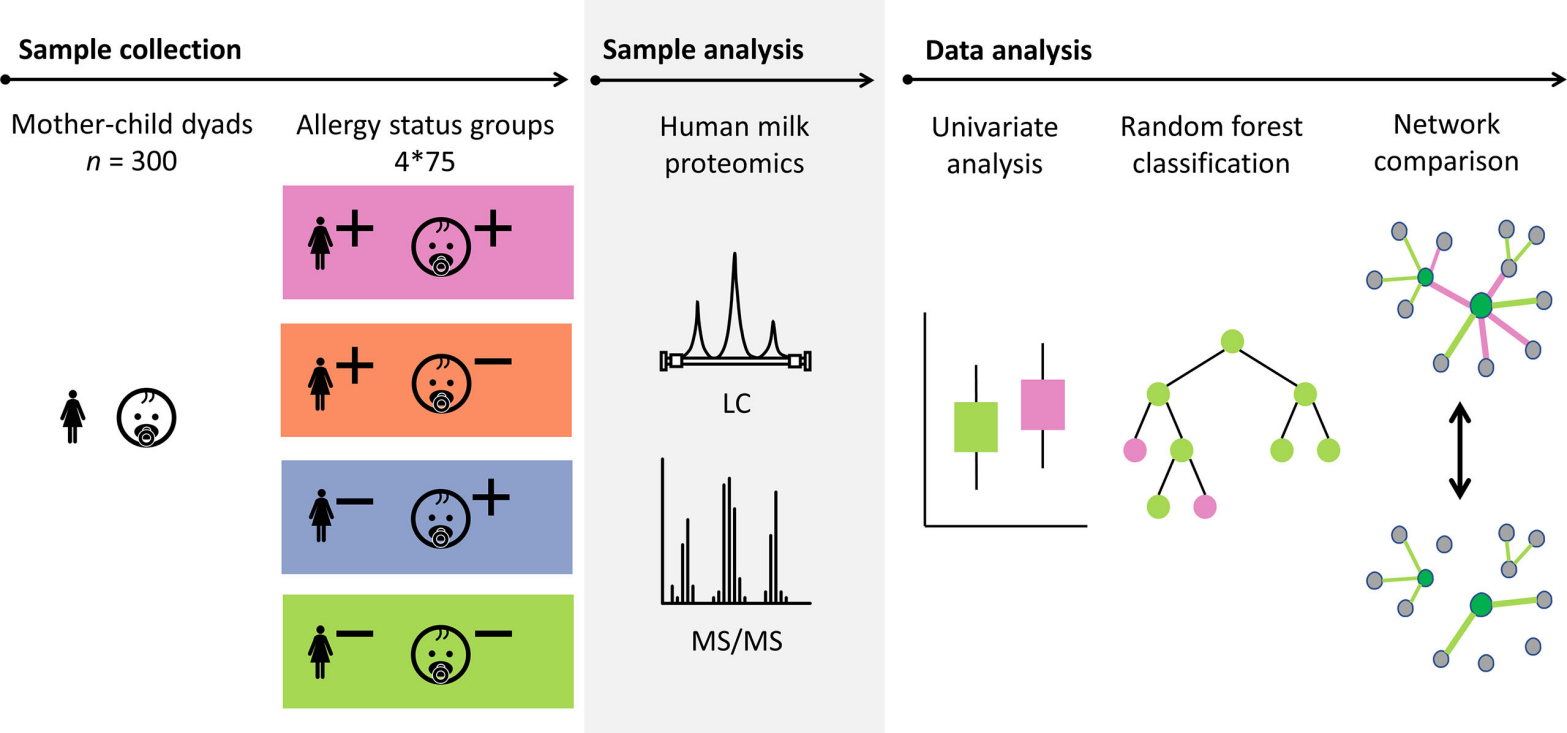
Schematic overview showing the sample set from the CHILD Cohort Study and the approach that was used for the analysis of the data. Proteins in a selection of 300 human milk samples from mother-child dyads with different allergy status (+ indicates allergy, - indicates no allergy) were analyzed using mass spectrometry. The data analysis was carried out using univariate analysis, classification models, and network comparison. Probabilistic Context Likelihood of Relatedness on Correlation (PCLRC), differential connectivity, and Covariance Simultaneous Component Analysis (COVSCA) were used for the network comparisons.

The research to date has been mostly limited to targeted, assay-based protein analysis, with a small number of identified proteins. As a result, little is currently known about the relation between the complete human milk proteome and the allergy status of mother and child. We set out to investigate this, using human milk samples from a subset of the Canadian CHILD Cohort Study, a general population birth cohort (21). This subset included 300 mother-child dyads, equally distributed across four groups representing all possible combinations of allergy status of both mother and child. The human milk proteome of these samples was analyzed with a shotgun/bottom-up proteomics workflow, meaning that proteins were analyzed through the identification of peptides that are released from the protein through trypsin digestion. The resulting data was investigated using univariate analysis, exploratory multivariate analysis, classification models, and network analysis (see **Figure 1**).

**Figure 2:**
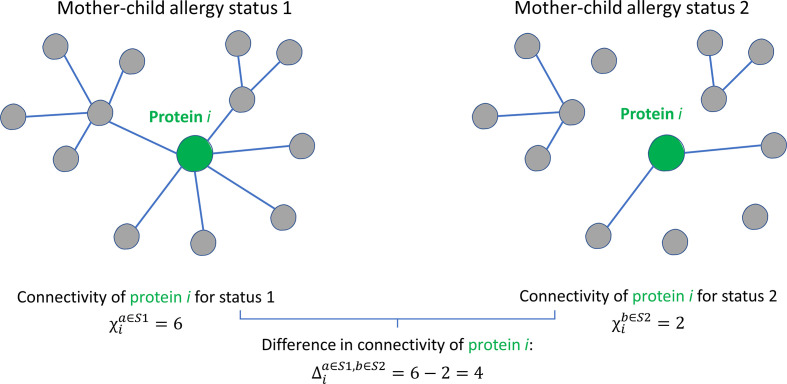
Graphical illustration of the concept of protein connectivity and differential connectivity. An unweighted protein-protein association network with 13 proteins is shown for two groups with different mother-child allergy status. Protein *i* is connected (a connection is represented by the existence of an edge) with a different number of proteins in the two groups. The connectivity *χ_i_* of protein *i* is given by the number of connecting edges: 6 for allergy status 1 and 4 for allergy status 2. The differential connectivity for protein *i* is given by $\Delta_{i}^{a\in S1,b\in S2}=\chi_{i}^{a\in S1}-\chi_{i}^{b\in S2}$ . This figure was adapted from Jahagirdar et al. (22).

Whereas in univariate analysis, the focus is on the abundance of the individual proteins, a systems biology approach with network analysis enables the consideration of interconnections between proteins. A protein network is a graphic representation of proteins (nodes) and their associations (edges or connections) expressed by a similarity measure such as correlation coefficient. The cause of protein-protein associations (i.e., why proteins correlate in abundance) can be due to different factors, and no hypothesis is set *a priori*. An example of a cause of such associations is that proteins have a shared location of synthesis, mechanism of transport, physical interaction, or molecular function, resulting in a correlation in their abundances. The protein connectivity (**Figure 2**), i.e., the extent to which proteins are associated with other proteins, can provide information on their interconnections and functioning as a whole (23, 24). Analysis of protein networks is essential in a thorough investigation of a possible relation between the milk proteome and a pathological condition such as allergy, because proteins are pivotal components in often interconnected biological processes and therefore often depend on other proteins in their functioning (25). Comparison of protein-protein association networks across conditions such as allergy status can be carried out using differential network analysis (**Figure 2**). Such analysis of differences in protein-protein associations (differential connectivity) can help to elucidate and better understand molecular mechanisms that might be affected by allergy status. Such information cannot necessarily be obtained from univariate analyses, which only focus on the abundance of individual proteins (26).

This study aimed to explore the relation between the complete human milk proteome on the one hand and both maternal allergy and child allergy development on the other hand, and is the first to undertake an untargeted analysis of the human milk proteome to examine this.

## Materials and methods

### Study design CHILD cohort

This study included a subset of *n* = 300 mother-child dyads originating from the CHILD cohort (https://www.childstudy.ca) (21). In the CHILD Cohort Study, pregnant mothers were recruited from the general population from four locations in Canada (Vancouver, Edmonton, Manitoba, and Toronto). The study was carried out following the Declaration of Helsinki, and local Human Research Ethics Boards approved the study protocols. All parents involved in the study provided written informed consent at enrollment.

The selection of the 300 mother-child dyads for the current study was made based on the allergy status of the mother and the child (**Figure 1**). Based on a 2x2 factorial design including allergy of both mother and child, four equal-sized groups (*n* = 75) were created (allergic mother and child, allergic mother and non-allergic child, non-allergic mother and allergic child, non-allergic mother and child). These 4 groups are later referred to as “mother-child allergy groups.” The groups were matched for lactation stage, maternal age, maternal BMI, secretor status, ethnicity, and infant sex.

### Definition of allergy

The definition of maternal allergy included at least one self-reported diagnosis of allergic disease, including asthma, food allergy, hay fever, or skin allergy, at the time of enrollment during pregnancy.

The definition of child allergy included atopic sensitization (1 or 3 years of age) with one or more of the following: atopic dermatitis (1 or 3 years of age), recurrent wheezing (1 year of age), asthma (3 years of age), rhinitis (3 years of age), or food allergy (3 years of age). Atopic sensitization was determined using standardized skin prick tests, including six inhalant allergens (*Alternaria alternata*, cat hair, dog epithelium, house dust mites [*Dermatophagoides pteronyssinus* and *Dermatophagoides farinae*], and German cockroach)) and four food allergens (bovine milk, egg, peanut, and soybean). According to the criteria described by Williams et al. (27), atopic dermatitis was assessed by pediatricians of the CHILD study. At three years of age, the CHILD study physician made a careful assessment of the child’s clinical history. Diagnoses recorded as “yes” and “possible” were considered positive for the purpose of defining whether the child had any of asthma, allergic rhinitis, food allergy, or atopic dermatitis. A detailed description of the assessments of allergic sensitization and diseases has been given before (28).

### Sample collection

Milk samples were collected according to the CHILD protocol (29). In short, foremilk and hindmilk samples were collected from several feedings during a day and were pooled to minimize within feed variation and diurnal variation. Samples were collected between 6 and 35 weeks post-partum (median = 15.6 weeks, interquartile range (IQR) = 4.6). Samples were stored at 4°C in the home refrigerator and within 24 hours picked up and transported on ice to the CHILD laboratory. There, samples were aliquoted and stored until further analysis at -80°C. Further transport of the samples was done on dry ice.

### Sample preparation

Skimmed milk was obtained by centrifugation at 10,000*g* and 4°C for 30 minutes. Then, skimmed milk was again centrifuged at 1,000*g* and 4°C for 10 minutes to remove any remaining lipids. The protein concentration was determined with the Pierce bicinchonic acid (BCA) assay (Thermo Scientific, Waltham, MA), and samples were diluted in 100 mM Tris to a concentration of 1 *µ*g/*µ*L protein. The diluted skimmed milk samples were prepared with filter-aided sample preparation (FASP) for protein analysis in randomized order as previously described (30).

In addition to the samples from the CHILD Cohort Study, aliquots of a pooled human milk sample were added as controls to check for technical variation. This sample comprised multiple aliquots of pooled human milk samples from the Dutch Human Milk Bank (Amsterdam, The Netherlands).

### LC-MS/MS analysis

Trypsin digested proteins were analyzed with LC-MS/MS as described before, with minor adjustments (31). In short, 1.5 - 4 *μ*L of tryptic peptide solution was loaded onto a 0.10 × 250 mm ReproSil-Pur 120 C18-AQ 1.9 *μ*m beads analytical column (prepared in-house) at 825 bar. A gradient from 9 to 34% acetonitrile in water with 0.1% formic acid in 50 min (Thermo nLC1000) was used. Full scan FTMS spectra were obtained using a Q-Exactive HFX (Thermo electron, San Jose, CA, USA) in positive mode between 380 and 1400 *m/z*.

The 25 most abundant positively charged peaks (2–5) in the MS scan were fragmented (HCD) with an isolation width of 1.2 *m/z* and 24% normalized collision energy. MSMS scans were recorded in data-dependent mode with a threshold of 1.2 × 10^5^ and 15 s exclusion for the selected *m*/*z* ± 10 ppm. Samples were analyzed with a technical replicate added randomly to each 7 injections.

### Data processing

The Andromeda search engine of the MaxQuant software v1.6.17.0 (32) was used to analyze the raw LC-MS/MS data. For this, a database was created by an initial MaxQuant run using the full human proteome (downloaded from UniProtKB on 20-01-2021, *n* = 194,237) (33). Protein identifiers obtained as identification from this initial run were used to create a human milk database for a second run (*n* = 24,175), in which also a cow milk protein (*n* = 1,006) and an allergen protein database (*n* = 721) were added, as described before (18).

In MaxQuant, digestion specificity was set to Trypsin/P, with maximally 2 missed cleavages. A fixed propionamide modification was set for cysteines and variable modifications for acetylation of the peptide N-term, deamidation of the side chains of asparagine and glutamine, and oxidation of methionine, with a maximum of five modifications per peptide were set.

Label-free quantification (LFQ) was used to obtain protein abundances. Per identified protein group, a leading protein was selected as described before (18) and proteins were manually annotated with keywords using the UniProtKB database (33) (accession date: 21-02-2022). Non-human sequences were only included if they had an identification score >80 and if there was no match with any human protein. The Peptide Match service of the online Protein Information Resource (34) was used to check for matches with human proteins. This service uses an up-to-date UniProtKB database and sequences were matched to this database without isoforms, where leucine and isoleucine were treated as equivalent.

The mass spectrometry proteomics data have been deposited to the ProteomeXchange Consortium *via* the PRIDE (35) partner repository with the data set identifier PXD034806. Sample metadata can be made available upon request. Requests can be submitted *via* email to child@mcmaster.ca.

### Statistical methods

#### Missing data

In dealing with the missing values in the proteomics data, identifications were first filtered with the requirement that proteins had a minimum of 25 valid values in at least one of the four sample groups. In practice this resulted in a minimum of 49 and a median of 209 valid values. This way of filtering the data prevented the removal of proteins that had only valid values in one of the four sample groups. Following this, the remaining missing values were imputed using the GSimp package (36). This Gibbs sampler-based algorithm imputes missing values with the assumption that missing values are not at random (MNAR) and left-censored.

#### Univariate analysis

The Kruskal-Wallis test was applied to deduce differences in protein abundance between the milk from mothers in the different mother-child allergy status groups (37). Resulting *p*-values were corrected for multiple testing using Benjamini-Hochberg correction (38). After correction, an adjusted *p*-value<0.05 was considered significant. Dunn’s multiple comparison test (39) was applied to determine differences between specific groups and also these *p*-values were corrected for multiple hypothesis testing using Benjamini-Hochberg correction.

#### Principal component analysis

For unsupervised data exploration, Principal Component Analysis (PCA) (40) was applied on the 300 × 687 data matrix (samples × proteins), using the FactoMineR package for R (41). This method enabled investigation of the data structure and the possible presence of patterns in protein abundance that cause differentiation between samples from groups with different allergy status. Data was scaled to unit variance before analysis.

#### Random Forest modeling

Random Forest (42) classification models were built using the R package “randomForest” (43) as described before (44). Six different models were built to discriminate between the different mother-child allergy groups, covering all pairwise comparisons of allergic/non-allergic mothers with allergic/non-allergic children. The significance of the reported results was assessed with a permutation test using 1000 permutations.

#### Network inference and analysis

##### Probabilistic context likelihood of relatedness on correlation (PCLRC)

For a more complete investigation of the proteome, relationships among proteins need to be considered. Such relationships between proteins can be captured using an index of association like a correlation coefficient (22). Protein-protein association networks were built using the Probabilistic Context Likelihood of Relatedness on Correlation (PCLRC) algorithm (45). This algorithm provides a robust estimation of correlation, using resampling and a modified version of the Context Likelihood of Relatedness (CLR) algorithm (46) to remove nonsignificant background correlations. A graphical representation of the pipeline used for the generation of the protein-protein association networks was provided by Saccenti et al. (45).

With resampling (*n* iterations = 1000), 75% of each dataset was randomly selected and subjected to the CLR algorithm. This process resulted in a matrix with a probabilistic measure *p_ij_* for each correlation between proteins r_*ij*_, where *i* and *j* indicate the *i*-th and *j*-th protein in the Spearman correlation matrix. With this resampling protocol, the likelihood of each observation is obtained. The output, a probabilistic network, contains estimates of how probable the association between any two proteins is. Correlations were retained if *p_ij_ >*0.99 and all other correlations were replaced with 0.


$$r_{ij}=\{\begin{array}{cc} r_{ij} & if\;p_{ij}\geq 0.99\\ 0 & if\;p_{ij}<0.99 \end{array}$$

Networks were built for each different mother-child allergy group, resulting in a total of 4 protein networks. The connectivity of a protein *i* in network *a* with mother-child allergy status *S* is defined according to:


$$\chi_{i}^{a\in S}=\left(\sum_{j=1}^{J}\left|r_{ij}\right|\right)-1$$

The differential connectivity (**Figure 2**) between two networks *a* and *b*, with different mother-child allergy statuses *S1* and *S2*, is calculated by:


$$\Delta_{i}^{a\in S1,b\in S2}=\chi_{i}^{a\in S1}-\chi_{i}^{b\in S2}$$

All *p*-values for differential connectivity were adjusted for multiple testing with Benjamini-Hochberg correction (38). Significant differential connectivities (*p<*0.05) were considered for further analysis and interpretation.

##### Covariance simultaneous component analysis (COVSCA)

To explore comprehensively the (dis)similarity among the protein association networks, Covariance Simultaneous Component Analysis (COVSCA) was used (47). With this approach, differences and commonalities between the different networks can be modeled.

In comparing networks with COVSCA, each network becomes a point in the component space. Thus, the method enables a representation and visualization of multiple networks in a way that is similar to PCA. Points (protein association networks of different sample groups) that are close to each other in the R-dimensional space share similar characteristics, i.e., similar correlation patterns between protein abundances. Furthermore, the loadings of the components give the relative contribution of each protein in shaping the observed network differences.

COVSCA, initially developed for modeling multiple covariance matrices at the same time, can also be used for the adjacency matrices resulting from PCLRC. The *K* matrices are modeled as a combination of low dimensional prototypes (*L*<< *K*):


$$S_{k}=\sum_{l=1}^{L}c_{kl}Z_{l}Z_{l}^{T}$$

In this, *c_kl_* ≥ 0(*l* = 1,2,…,*L*) are weight coefficients, and $Z_{l}Z_{l}^{T}$ are prototypical symmetric matrices consisting of loading ***Z*** of size *J* × *R_l_* that hold simultaneously for all *S_k_*.

Two rank-1 prototype matrices were used to fit the model, resulting in one set of loadings per component. This fit was chosen as the best compromise between goodness off it (74%) and the complexity of the COVSCA model (rank and number of the prototypical matrices). COVSCA loadings were transformed to *z*-scores and loadings with *z* > |2| were further investigated.

#### Overrepresentation analysis

The GORILLA (Gene Ontology enRIchment anaLysis and visuaLizAtion tool) (http://cbl-gorilla.cs.technion.ac.il/) tool (48) was used for overrepresentation analysis of gene ontology (GO) annotations in proteins that were differentially connected. The tool was used in two list mode where all proteins identified in the current study were used as background set. All *p*-values reported were corrected with Benjamini-Hochberg correction (38), and considered significant if *p<*0.05.

## Results

Proteomic analysis of all samples led to a total of 1690 identified proteins before filtering on missing values. After filtering these proteins on the requirement of being identified ≥ 25 times in at least one of the four mother-child allergy groups, 687 proteins remained for further data analysis. In this filtered dataset, the number of identified proteins per sample ranged between 242 and 636 (median = 480). The major milk proteins *α*-lactalbumin, albumin, lactoferrin, *β*-casein, and *α*_s1_-casein, were in all analyzed samples among the 15 most abundant proteins. A complete overview of the 687 identified proteins can be found in **Supplementary Table 1**.

### Univariate analysis

Differences in protein abundance between the different mother-child allergy groups were assessed with Kruskal-Wallis tests. After correction for multiple hypothesis testing, no significant differences were found among the four groups (**Table 1**). Kruskal-Wallis outcomes with uncorrected p < 0.05 were further assessed with Dunn’s *post-hoc* tests and subsequent correction for multiple testing, which resulted in 30 proteins that showed a difference between the groups with corrected p < 0.05 (**Table 1**).

**Table 1:** Results of univariate analysis (Kruskal-Wallis) with subsequent *post-hoc* test (Dunn’s) for the comparison of protein abundance in milk from allergic (M+) and non-allergic (M-) mothers, with children who developed an allergy (C+) and did not develop an allergy (C-) in the CHILD Cohort Study.

					Comparison			
Leading protein	UniProt ID	Keyword	*p*-value^a^	Adjusted *p*-value^a^	Group 1	Group 2	Adjusted *p*-value^b^	Trend
Sodium-dependent phosphate transport protein 2B	O95436	Transport	0.008	0.867	M-/C-	M-/C+	0.014	⇑
V2-7 protein	A2MYD4	Immunoglobulin	0.029	0.957	M-/C-	M-/C+	0.041	⇓
IGL c1836-light	A0A5C2G0A5	Immunoglobulin	0.014	0.957	M-/C-	M-/C+	0.019	⇓
IGL c2315-light	A0A5C2G2Y4	Immunoglobulin	0.031	0.957	M-/C-	M-/C+	0.037	⇓
IGH + IGL c632-heavy	A0A5C2GC20	Immunoglobulin	0.012	0.944	M-/C-	M-/C+	0.008	⇓
IG c662-heavy	A0A5C2GE75	Immunoglobulin	0.002	0.453	M-/C-	M-/C+	0.002	⇓
IG c326-heavy	A0A5C2GF50	Immunoglobulin	0.003	0.453	M-/C-	M-/C+	0.001	⇓
IG c849-heavy	A0A5C2GF92	Immunoglobulin	0.020	0.957	M-/C-	M-/C+	0.025	⇓
IG c279-heavy	A0A5C2GLS6	Immunoglobulin	0.011	0.944	M-/C-	M-/C+	0.008	⇓
IG c1707-heavy	A0A5C2GYK2	Immunoglobulin	0.002	0.453	M-/C-	M-/C+	0.001	⇓
Methyltransferase-like protein 9	H3BM54	Methyltransferase	0.001	0.453	M-/C-	M-/C+	0.028	⇓
Delta-1-pyrroline-5-carboxylate synthase	P54886	Proline biosynthesis	0.026	0.957	M-/C-	M-/C+	0.039	⇓
Prosaposin variant	Q53FJ5	Lipid metabolism	0.028	0.957	M-/C-	M-/C+	0.018	⇓
Immunoglobulin heavy	Q9NPP6	Immunoglobulin	0.032	0.957	M-/C-	M-/C+	0.036	⇓
IgG L chain	S6BAR0	Immunoglobulin	0.023	0.957	M-/C-	M-/C+	0.026	⇓
N90-VRC38.07 heavy	A0A1W6IYI6	Immunoglobulin	0.033	0.957	M-/C-	M+/C-	0.019	⇓
V2-7 protein	A2MYD4	Immunoglobulin	0.029	0.957	M-/C-	M+/C-	0.041	⇓
IGL c1836-light	A0A5C2G0A5	Immunoglobulin	0.014	0.957	M-/C-	M+/C-	0.019	⇓
IG c326-heavy	A0A5C2GF50	Immunoglobulin	0.003	0.453	M-/C-	M+/C-	0.043	⇓
IG c849-heavy	A0A5C2GF92	Immunoglobulin	0.020	0.957	M-/C-	M+/C-	0.025	⇓
Nephronectin	Q6UXI9	Calcium binding	0.032	0.957	M-/C-	M+/C-	0.021	⇑
Phospholipid hydroperoxide glutathione peroxidase	P36969	Peroxidase	0.028	0.957	M-/C-	M+/C+	0.021	⇓
Sodium-dependent phosphate transport protein 2B	O95436	Transport	0.008	0.867	M-/C-	M+/C+	0.014	⇓
Hornerin	Q86YZ3	Keratinization	0.024	0.957	M-/C-	M+/C+	0.020	⇑
Galectin-3-binding protein	Q08380	Cell adhesion	0.037	0.999	M-/C+	M+/C-	0.023	⇑
IGL c2315-light	A0A5C2G2Y4	Immunoglobulin	0.031	0.957	M-/C+	M+/C-	0.037	⇑
IG c662-heavy	A0A5C2GE75	Immunoglobulin	0.002	0.453	M-/C+	M+/C-	0.017	⇑
Methyltransferase-like protein 9	H3BM54	Methyltransferase	0.001	0.453	M-/C+	M+/C-	0.034	⇑
Alpha-S1-casein	A0A0J9YVR3	Milk protein	0.035	0.993	M-/C+	M+/C+	0.043	⇓
IG c662-heavy	A0A5C2GE75	Immunoglobulin	0.002	0.453	M-/C+	M+/C+	0.017	⇑
Methyltransferase-like protein 9	H3BM54	Methyltransferase	0.001	0.453	M-/C+	M+/C+	0.000	⇑
Ectonucleoside triphosphate diphosphohydrolase 3	O75355	Hydrolase	0.022	0.957	M-/C+	M+/C+	0.040	⇓
Protein FAM3C	Q92520	Cytokine	0.008	0.867	M-/C+	M+/C+	0.009	⇑
IgG L chain	S6BAR0	Immunoglobulin	0.023	0.957	M-/C+	M+/C+	0.026	⇑

a from Kruskal-Wallis tests.

b from Dunn’s post-hoc tests.

The trend indicates higher (⇑) or lower (⇓) abundance in the first group in the comparison. Listed are all proteins with uncorrected p-value< 0.05 (Kruskal-Wallis) and corrected p-value< 0.05 (Dunn’s), sorted by the mother-child allergy groups in the comparison.

**Figure 3:**
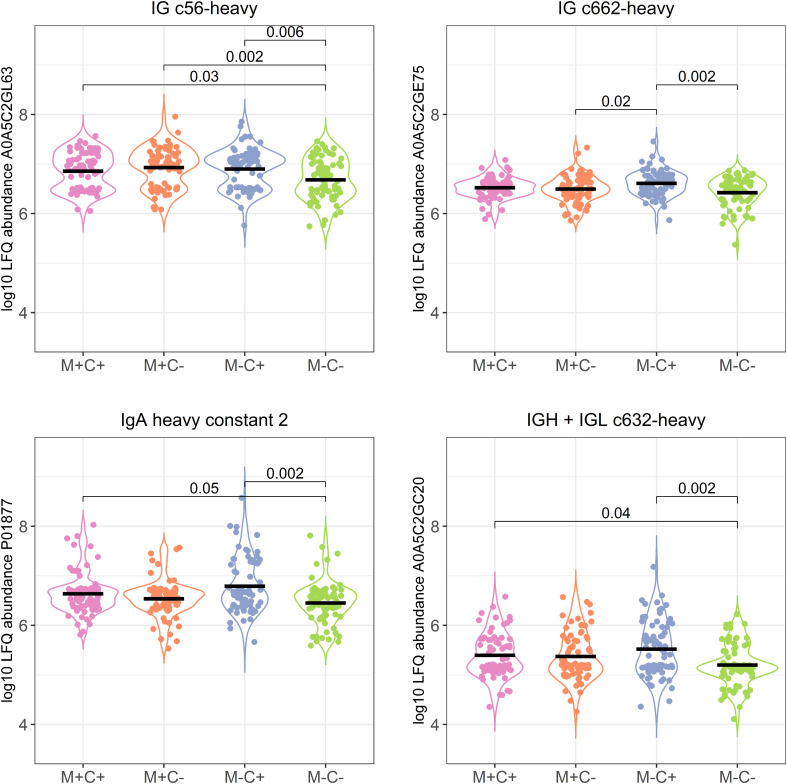
Violin plots visualizing the differences in abundance of the 4 most significantly different immunoglobulin (Ig) chains between the different allergy status groups from the CHILD Cohort Study. Differences between groups are indicated with *p*-values from Dunn’s *post-hoc* tests, and means of each group are shown with black, horizontal lines. In the labeling of the groups, M indicates mother, C indicates child, + indicates allergy, and - indicates no allergy.

Most of these differences (*n* = 17) were found between the non-allergic group (M-C-) and the group where only the child ultimately developed an allergy (M-C+). Proteins that differed between these groups were primarily Ig chains (15 out of 17) and were mostly higher in abundance in the group where the mother was non-allergic and the child developed an allergy (**Figure 3**). Additionally, 3 of these Igs show also a higher abundance in milk from allergic mothers with children who did not develop an allergy.

**Figure 4:**
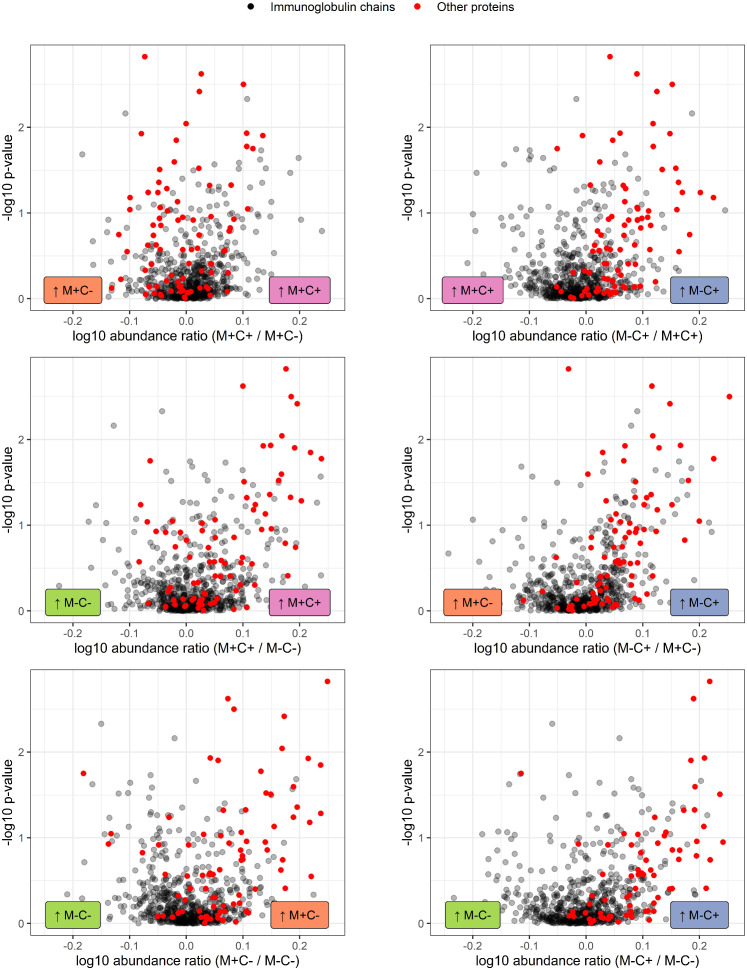
Volcano plots visualizing the trend in immunoglobulin abundances in milk from different mother-child allergy status groups from the CHILD Cohort Study. Each data point represents one protein, with on the x-axes the ratio of the means of the log10 transformed label-free quantification (LFQ). Immunoglobulin-related proteins are represented by red and other proteins with grey dots. Colored labels on left and right side of x = 0 indicate in which mother-child allergy status group the mean abundance of the respective proteins is higher. In the labeling of the groups, M indicates mother, C indicates child, + indicates allergy, and - indicates no allergy. A trend can be observed that most immunoglobulin-related proteins are higher in abundance in the group where the mother is non-allergic and the child ultimately develops an allergy.

Further investigation of all identified Ig proteins showed that the mean abundance of these proteins is generally lower in the groups where mother, child or both are allergic, when compared to the non-allergic group (**Figure 4**). This effect is clearest in the comparison of the group where only the child developed an allergy with the group where both mother and child are nonallergic. Out of 83 Ig proteins, 77 have a mean abundance that is higher in the group where the child developed an allergy.

### Non-human proteins

In the current study, several non-human proteins were identified (*n* = 11), including albumin from dog, horse, and cat, as well as bovine *α*_s1_-casein and BLG (**Table 2**). However, the majority of these proteins were only found with few tryptic peptides in a low number of samples and filtered out before further data analysis. Additional non-human proteins of potential interest that were included in the database, but not identified in any samples, include allergens from, for example, peanut, egg, and house dust mite.

**Table 2:** Identified non-human tryptic peptides in human milk samples from the CHILD Cohort Study (*n* = 150 allergic mothers and 150 non-allergic mothers).

Sequence	UniProt ID	Leading protein	Organism	Identified in *n* (%) samples from allergic mothers	Identified in *n* (%) samples from non-allergic mothers	Identification score^b^
KQTALVELLK	P49822	Albumin	Bos taurus (Bovine)	11 (7)	18 (12)	87.3
LVNELTEFAK	P02769	Albumin	Bos taurus (Bovine)	100 (67)	103 (69)	125.0
gEKVNELSK	P02662	*α*_s1_-casein	Bos taurus (Bovine)	2 (1)	3 (2)	149.7
HIQKEDVPSER	P02662	*α*_s1_-casein	Bos taurus (Bovine)	4 (3)	1 (1)	93.6
IDALNENK	P02754	*β*-lactoglobulin	Bos taurus (Bovine)	12 (8)	11 (7)	89.8
LISVDTEHSNIYLQNGPNR	F1N076	Ceruloplasmin	Bos taurus (Bovine)	28 (19)	32 (21)	203.6
MFTTAPDQVDKENEDFQESNK	F1N076	Ceruloplasmin	Bos taurus (Bovine)	2 (1)	3 (2)	88.0
SSQDLQPR	Q0P5H7	Probable arginine–tRNA ligase	Bos taurus (Bovine)	32 (21)	32 (21)	81.4
FPKADFAEISK	P49822	Albumin	Canis lupus familiaris (Dog)	4 (3)	4 (3)	90.2
LVNEVTEFAKK	Q5XLE4	Albumin	Equus caballus (Horse)	147 (98)	139 (93)	124.1
AEFAEISK	P49064	Albumin	Felis catus (Cat)	73 (49)	82 (55)	84.9
AFKAWSVAR	P49064	Albumin	Felis catus (Cat)	100 (67)	109 (73)	98.3
EVCKNYQEAK	P49064	Albumin	Felis catus (Cat)	94 (63)	95 (63)	96.5
YICENQDSISTK	P49064	Albumin	Felis catus (Cat)	17 (11)	11 (7)	85.4

^a^ Score from the MaxQuant output indicating the quality of the identification of the peptide. A higher score represents a better identification.

### Multivariate exploratory analysis

**Figure 5:**
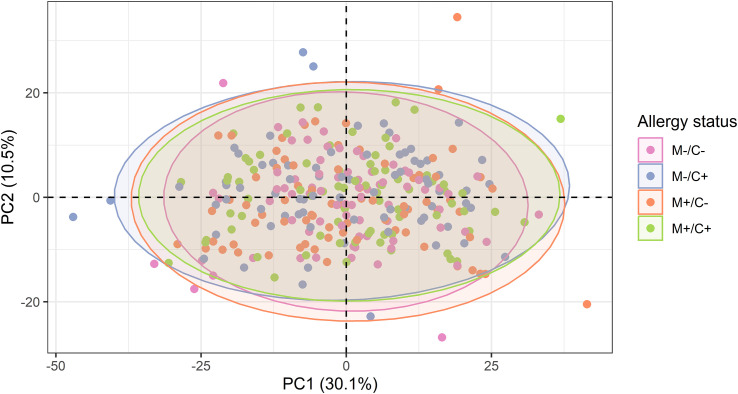
Scatter plot of principal component analysis (PCA) representing the human milk protein profile of mother-child dyads from the CHILD Cohort Study. Each point represents one dyad. No obvious differences can be observed among protein profiles of different mother-child allergy groups using this method.

To explore whether patterns in the abundance of proteins allow a differentiation of the different mother-child allergy status groups, PCA was performed. The visualization of all samples using the first two components of the PCA shows that there is no separation between the groups of different allergy status of mother and child (**Figure 5**), suggesting no major global differences between the protein profiles of these four groups.

### Prediction of allergy status using Random Forest models

Random Forest classification was used to discriminate the samples of the different mother-child allergy groups based on the milk protein profile. Two-group models were built for all combinations of maternal allergy status and child allergy status. From the results shown in **Table 3**, it can be noted that all classification models have low discriminating power and that it was therefore not possible to discriminate between the groups. The best accuracy, 60% which would still be considered “poor,” was obtained for the model that discriminates between the group where only the child developed an allergy and the group where both mother and child were non-allergic. Together, this indicates that difference in allergy status is only to a limited extent reflected in the abundance of proteins in human milk, as was also shown by the univariate analysis.

**Table 3:** Outcome of Random Forest models on human milk proteins for the discrimination of groups with different allergy statuses from the CHILD Cohort Study.

Comparison					
Group 1	Group 2	Accuracy (%), (*p*-value)	Specificity (%), (*p*-value)	Sensitivity (%), (*p*-value)	AUROC, (*p*-value)
M+/C+	M+/C-	46.7 (0.62)	49.3 (0.39)	44.0 (0.76)	51.7 (0.81)
M+/C+	M-/C+	50.7 (0.34)	45.3 (0.62)	56.0 (0.10)	53.2 (0.63)
M+/C+	M-/C-	50.0 (0.38)	53.3 (0.18)	46.7 (0.60)	51.1 (0.86)
M+/C-	M-/C+	49.3 (0.41)	50.7 (0.29)	48.0 (0.52)	51.4 (0.84)
M+/C-	M-/C-	54.7 (0.14)	56.0 (0.11)	53.3 (0.23)	50.6 (0.94)
M-/C+	M-/C-	60.0 (0.01)	58.7 (0.05)	61.3 (0.01)	58.6 (0.22)

Comparisons of the groups are labelled according to allergy status, with allergic (M+) and non-allergic (M-) mothers, and allergic (C+) and non-allergic (C-) children.

### Network analysis

Next, differential network analysis was applied to investigate whether maternal allergy status or the development of allergy in the child is reflected in the milk protein profile in more complex ways.

#### Network inference

**Figure 6:**
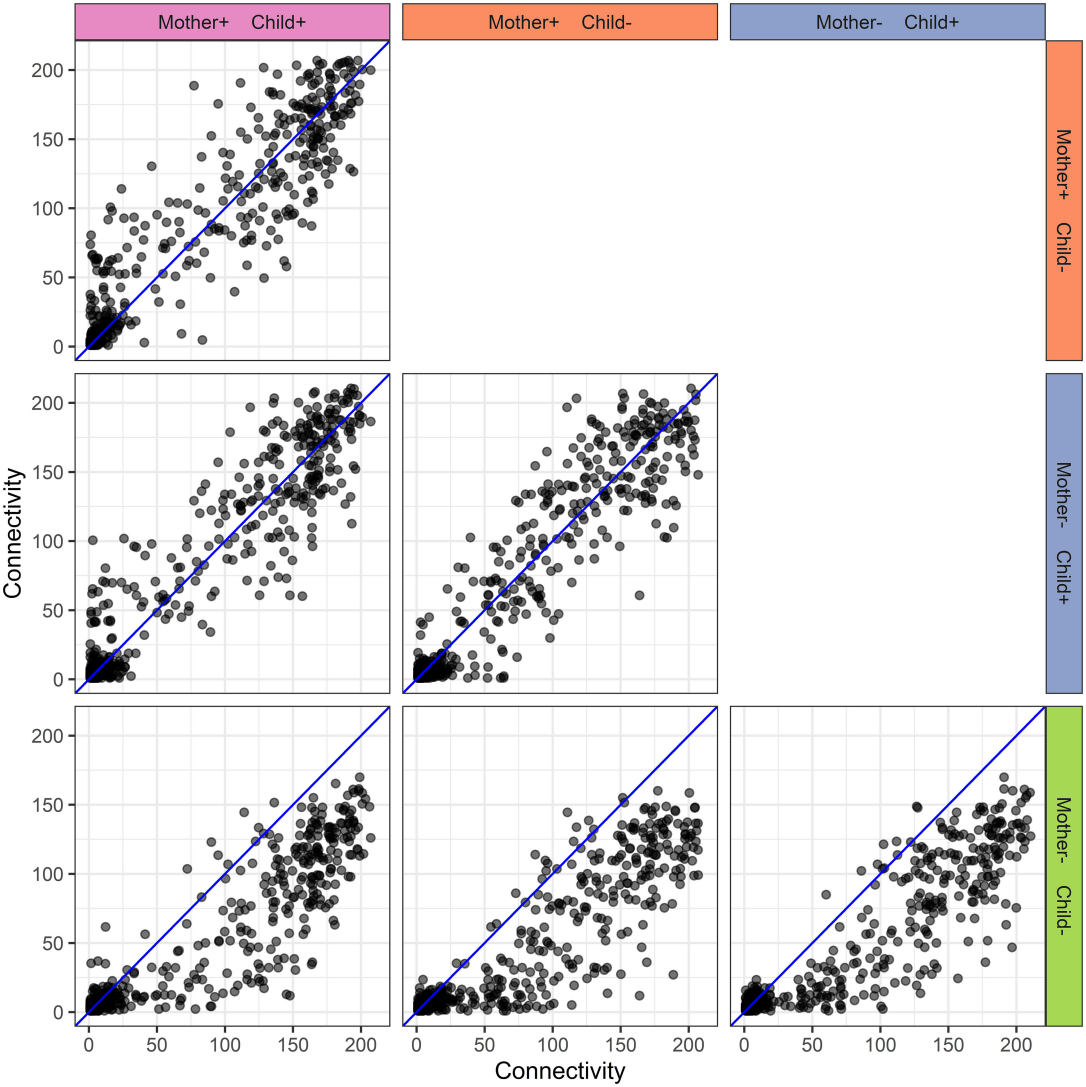
Human milk protein connectivity in the different mother-child allergy groups from the CHILD Cohort Study. Each subplot represents a pairwise comparison of protein connectivity in two mother-child allergy groups and each dot represents a single protein. Protein connectivity is obtained from the adjacency matrices build with the PCLRC algorithm and all groups are compared with one another in each subplot. In the labeling of the groups, + indicates allergy and - indicates no allergy. The group in which both mother and child are non-allergic shows a distinct connectivity pattern with an overall lower connectivity of the proteins.

The protein-protein association networks (**Supplementary Figure 1**) of the different mother-child allergy status groups were used to calculate the connectivity of each protein in each mother-child allergy group. The PCLRC algorithm retained mostly positive associations and connectivity represents the number of associations per protein. A comparison of the protein connectivity is visualized in **Figure 6**. What can be observed from this figure is a pattern that for the groups where at least either mother or child is allergic, there is stronger interconnectivity between milk proteins when compared to the group where both mother and child are non-allergic.

To investigate this pattern further, proteins with differential connectivity > 50 were selected for further investigation (**Supplementary Table 1**). These proteins had the largest differences in connectivity among the four different groups and were selected for further functional analysis, to determine possible functional consequences of the differences between the networks. The selection resulted in 173, 171, and 153 proteins for the comparison of the group with non-allergic mother and child with respectively (*i*) allergic mother and non-allergic child, (*ii*) non-allergic mother and allergic child, and (*iii*) allergic mother and child groups. From these proteins, 95 proteins occurred in all three selections, showing a similarity in differential connectivity. Interestingly, GO overrepresentation analysis of these proteins showed a significant overrepresentation of proteins involved in translation initiation (*p* = 1.08 × 10^-15^). This overrepresentation is due to 23 ribosomal proteins and translation elongation factor EEF1A1P5.

None of the differentially connected proteins showed a difference in abundance between the different mother-child allergy groups with univariate analysis, indicating the complementarity of these two approaches.

#### Network modeling

**Figure 7:**
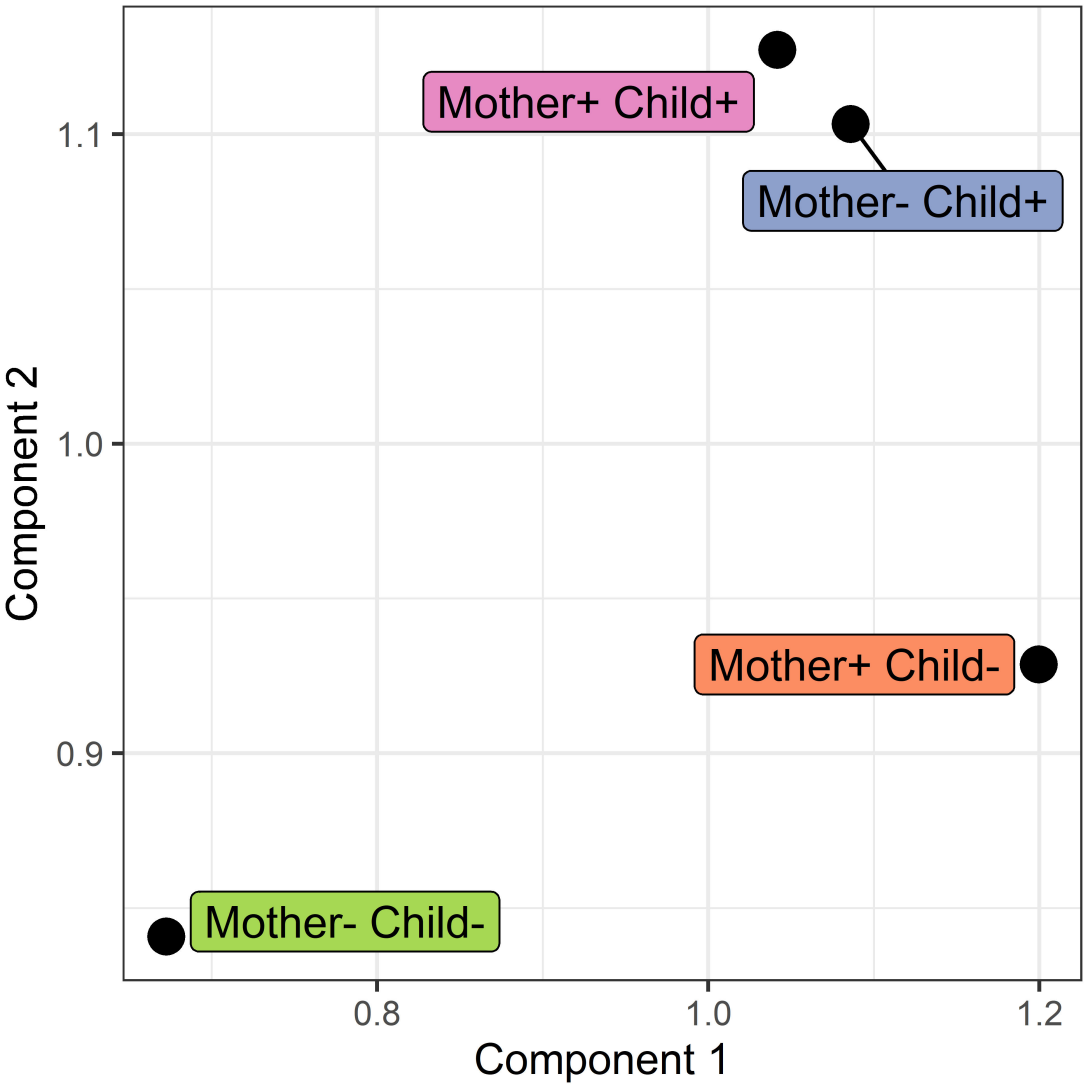
Score plot of the COVSCA model for the protein correlation network obtained using PCLRC of different groups based on maternal and child allergy status in the CHILD Cohort Study. Each point represents a protein-protein association network of one mother-child allergy group (+ indicates allergy, and - indicates no allergy). Protein importance for each component is shown in **Figure 8**. The groups with children who ultimately developed an allergy show similarities, whereas all the other groups show dissimilarities in correlation patterns.

In addition to pairwise comparison of networks, a simultaneous comparison was carried out using COVSCA. With COVSCA, similarities and differences in protein-protein correlation patterns can be analyzed for a set of networks. In the visualization of the results of COVSCA, each network is a data point in the component space (see **Figure 7**). In this comparison, the networks of the four different groups were compared. From this, it can first be observed that the group with both non-allergic mothers and non-allergic children shows differences in correlation patterns with all other groups. Both Component 1 and 2 account for the separation between these groups. Second, the group where only mothers are allergic shows a difference in network correlation patterns with the non-allergic group on Component 1. Thirdly, groups comprising children who developed an allergy show similarities in correlation patterns on both components.

To investigate these observations further, the loadings of the COVSCA model with *z* > |2| were examined. These loadings represent the proteins that contributed the most to the difference in correlation patterns between the different networks.

**Figure 8:**
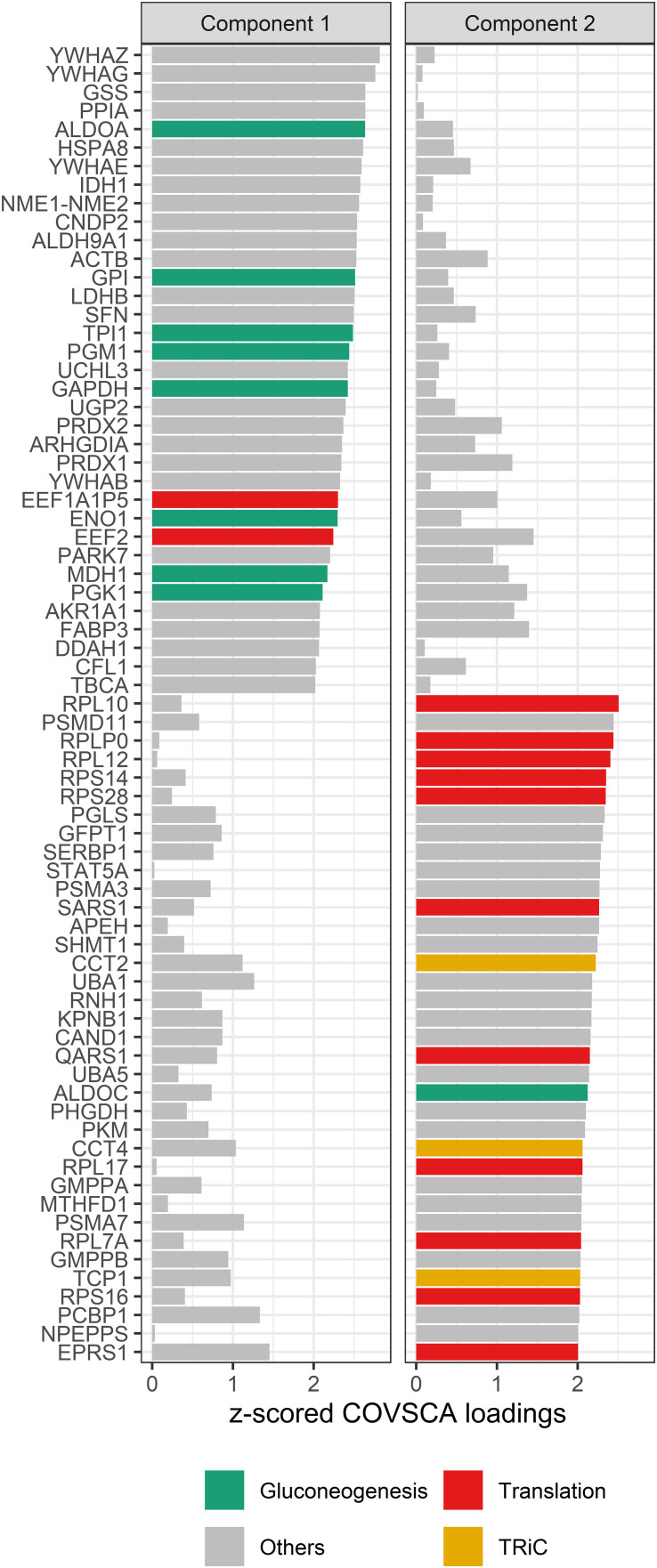
COVSCA loadings of the COVSCA model of different groups based on maternal and child allergy status in the CHILD Cohort Study. Loadings indicate the importance of each protein for the differences or similarities in correlation patterns observed in the COVSCA score plot (**Figure 7**). Proteins are labeled with gene IDs along the y-axis, and colors indicate shared gene ontology annotations. Among the proteins important for explaining the variability between the networks are proteins involved in gluconeogenesis, translation, and the tailless complex polypeptide 1 ring complex (TRiC).

The loadings for component 1 (see **Figure 8**) are overrepresented by proteins involved in gluconeogenesis (*p* = 0.0003), the synthesis of glucose. This component accounts for separation between the non-allergic group and the other three groups. The second component, which drives the separation of the groups on allergy status of the child, shows a significant overrepresentation of proteins involved in the positive regulation of DNA biosynthetic processes (*p* = 0.0013). This overrepresentation is mainly due to 5 members of the tailless complex polypeptide 1 ring complex (TRiC or CCT). In addition, several proteins involved in translation processes show differences in correlation patterns on this component.

## Discussion

We investigated the associations of human milk proteins with maternal and child allergy. Using univariate analysis, predictive modeling, and network analysis, several relevant differences and distinctive patterns were found between groups differing in allergy status.

### Differences in immunoglobulin abundances between groups with different allergy statuses

Several proteins showed differences in relative abundance when the different mother-child allergy groups were compared. Although none were statistically significant after traditional correction for multiple testing, this does not necessarily imply they are biologically insignificant. It is widely acknowledged that correction methods for multiple hypothesis testing can be too stringent for bottom-up proteomics data (49, 50) because each protein is represented by multiple tryptic peptides. Therefore, we reported both corrected and uncorrected *p*-values, and discuss the findings.

Most of the differences in protein abundance were found between non-allergic mothers with non-allergic children and the group where only the child developed an allergy. This result was also reflected in the accuracy of the Random Forest classification model for these two groups, which was the highest (60%) among all models. The differentially abundant proteins were mainly Ig variable domains. These results point to differences in specific Igs in milk consumed by children who ultimately develop an allergy, and these differences did not seem to be directly linked to the mother’s allergy status. This finding raises two important questions for future research: (*i*) why do these mothers secrete these specific Igs in higher abundance in their milk, and (*ii*) could the development of allergy in children be related to these Igs?

Regarding the first question, the findings in this study show that, regardless of maternal allergy status, milk for children who ultimately develop an allergy had higher abundances of specific Igs. Possibly, other factors that lead to allergy development in the child, such as health conditions, genetics, dietary patterns, or environmental exposures, also lead to higher abundance of Igs in the milk. Another possibility is that infants who would develop an allergy somehow cause higher abundance of specific Igs in the milk of the mother. Further research is required to explore these possibilities.

When it comes to the second question, there is contradicting evidence. It has been shown that higher abundance of specific Igs in human milk could help in the healthy development of the child’s immune system. For example, a study conducted by Ohsaki et al. (51) showed that ovalbumin-specific IgG immune complexes in human milk fed to mice induced tolerance. A study by Lupinek et al. (52) complements this by showing that allergen-specific IgG originating from cord blood or breast milk seemed to protect against allergic sensitization. Nevertheless, Järvinen et al. (16) showed that cow’s milk specific IgA levels in human milk did not correlate with the development of cow’s milk allergy in the child.

Unfortunately, more details on the function or specificity of the identified Ig variable domains are not available. A complete analysis of the sequence diversity of the antibody repertoire could be done with targeted approaches (53–55), but was outside the scope of the current study.

Notably, soluble CD14, a protein in human milk that may be protective against the development of food allergies (56, 57), was not different between the allergy groups in our study (uncorrected *p* = 0.43). This and other contradictions with prior studies could be related to our clinical definition of allergy. For example, in a previous study, significant differences were observed in comparing milk from mothers with house dust mite allergy and non-allergic mothers (58). These differences concerned especially protease inhibitors and apolipoproteins. We did not find these proteins to be different in abundance, which is possibly due to differences in the definition of allergy. Hettinga et al. (58) used a rather strict definition of house dust mite allergy, combined with high immunoglobulin E (IgE) levels in the blood and high environmental exposure to house dust mite, whereas we applied a more heterogeneous definition encompassing diagnosis of multiple allergic conditions.

Relatively few non-human proteins were identified in a low number of samples, and no apparent differences were observed between the different allergy groups (**Table 2**). Nevertheless, some studies have argued that non-human proteins, especially allergens, play an important role in allergy development (19, 59, 60). Data from several sources show that most of these proteins originate from the diet and especially from cow’s milk or cow’s milk products (18, 61). The difference between prior studies and the current study might be due to differences in, e.g., maternal consumption of dairy products. The presence of horse albumin in nearly half of the samples, is an intriguing finding. The respective sequence was identified before in human milk (18) and might originate from horse dander (62) or other products containing horse. Another explanation might be that the respective sequence is not unique for horse, although no evidence was found for this.

### Distinctive patterns of connectivity for groups with different allergy statuses

A particularly novel aspect of our study was the network analyses, which demonstrated distinctive association patterns between proteins when groups differing in allergy status were compared. These differences in the networks point to differences in pathway regulation being specific for one or more groups. Our most striking finding is the overall lower protein connectivity observed in the group where both mother and child are non-allergic. This overall difference in connectivity might reflect maternal lifestyle, environmental exposures, or health. For example, a recent study by Yan et al. (63) showed that disease-associated stress brought about the remodeling of protein pathways, leading to a proteome-wide increase in interaction strength and change in connectivity. Although such an increase in connectivity has not been described before regarding the human milk proteome and allergy, there is evidence showing that allergies are linked to systemic inflammation (64, 65). Such a state of systemic inflammation might, in turn, result in a change in protein connectivity in the human milk proteome.

Interestingly, in the COVSCA model, we observed that correlation patterns of proteins involved in gluconeogenesis were important for separation between the non-allergic group and the other three groups. The importance of these proteins in the separation points to differences in the regulation of glucose allocation to the mammary epithelial cells, which could reflect a competition between immune and epithelial cells for glucose, as it is known that during an immune response, immune cells need more glucose (66).

We also detected an overrepresentation of proteins involved in the translation machinery among the differentially connected proteins, which suggests a difference or dysregulation in translation machinery in allergic and non-allergic mothers with children who will develop an allergy. In addition, COVSCA loadings show different correlation patterns between these groups for proteins from the TRiC/CCT complex, which plays an essential role in protein folding and proteostasis (67). How these TRiC/CCT proteins and proteins from the translation machinery end up in the milk is not known, but they might originate from cells present in the milk (68). Their difference in connectivity among the different groups might then be due to, for example, different types of cells ending up in milk or different metabolic pathways being active in these cells. The latter would be in line with Calvano et al. (69), who found that in blood leukocytes from patients with systemic inflammation, there are dysregulations in, amongst others, elongation initiation factors and ribosomal proteins. This dysregulation could explain the stronger connectivity of the protein synthesis machinery in milk from allergic mothers, who possibly have a higher level of systemic inflammation. Nevertheless, stronger connectivity was also observed in milk from non-allergic mothers with children who would develop an allergy. No studies were found that could explain this observation, and further research should be undertaken to investigate and clarify this.

### Limitations and strengths

Although bottom-up proteomics has many advantages, it also has limitations. One of these is the dependence on a database; in other words, protein sequences not present in the database cannot be identified. This limitation poses a challenge, for example, in the identification of the variable regions of the Igs, of which many sequences are not available in databases. Another limitation is the large number of identifications resulting from these techniques, which requires stringent multiple hypothesis testing in classical univariate data analysis. Finally, although a relatively large sample size was used in the current study, it included considerable clinical heterogeneity in the definition of ‘allergy.’ It is possible that this clinical heterogeneity could have obscured the effect resulting from specific allergy phenotypes (e.g., food allergies or asthma) if these would have distinct associations with milk proteins.

In summary, this study set out to investigate the human milk proteome and its relations with both maternal allergy status and child allergy development. The results show trends in differential abundances of immune-related proteins between the mother-child allergy groups, suggesting possible variation in the immunological potential of human milk. However, an attempt to exploit these differences to build Random Forest classification models resulted in low predictive power. This outcome was confirmed with multivariate exploratory analysis which did not show differences in the data structure for the different groups. Interestingly, using a network approach, which enables investigation of protein-protein associations, significant differences were found among the different groups. The major finding was an overall stronger connectivity of proteins in the milk of allergic mothers and milk for infants who ultimately developed an allergy, showing that the allergy status of either mother, child, or both is reflected in the interconnectedness of the milk proteins. Collectively, these results show that network analysis complements univariate analysis, multivariate analysis, and classification models to reveal complex relationships between maternal-child allergy phenotypes and the human milk proteome. Specifically, the network analysis points to a difference in the regulation of translation processes and protein folding in the groups where the child ultimately developed an allergy, possibly reflecting the physiological state of the mother. Further research is warranted to investigate these associations and the implicated biological pathways to understand their possible functional role in allergy development and prevention.

## Data availability statement

The data presented in the study are deposited in the PRIDE repository, accession number PXD034806.

## Ethics statement

The studies involving human participants were reviewed and approved by Human Research Ethics Boards at McMaster University, the Hospital for Sick Children, and the Universities of Manitoba, Alberta, and British Columbia. The patients/participants provided their written informed consent to participate in this study.

## Author contributions

KH and MA conceived the original idea and project planning. KH, PD, and MA contributed to the study design. MA, PM, TM, ES, PS, and ST organized the clinical study and actively recruited patients. PD and SB carried out laboratory work and experiments. PD and ES performed the data analysis. PD drafted the manuscript. Review and editing was done by KH, ES, and MA. All authors contributed to the article and approved the submitted version.

## Funding

This research was funded by the Netherlands Organization for Scientific Research (NWO-TTW) (project number 15299). The Canadian Healthy Infant Longitudinal Development study was supported by both the Canadian Institutes of Health Research (CIHR) and the Allergy, Genes and Environment (AllerGen) Network of Centres of Excellence.

## Acknowledgments

The authors dedicate the paper to the bright memory of Jacques J.M. Vervoort, a friend and recognized scientist, who passed away in July 2021. JV contributed substantially to the conception and design of this study. The authors thank Bassel Dawod for his help with the samples and the method section, and thank the CHILD Cohort Study (CHILD) participant families for their dedication and commitment to advancing health research. In addition, we are grateful to everyone without whom this study could not have been completed, including all members, and staff of the CHILD Cohort Study. These include research staff, administrative staff, volunteers, lab technicians, statisticians, and clinical staff at the following institutions: McMaster University, University of Manitoba, University of Alberta, University of Toronto, and University of British Columbia.

## Conflict of interest

MA holds a Tier 2 Canada Research Chair in the Developmental Origins of Chronic Disease at the University of Manitoba and is a Fellow in the Canadian Institutes for Advanced Research (CIFAR) Humans and the Microbiome Program. She receives research funding from the Canadian Institutes of Health Research, Research Manitoba, the Canada Foundation for Innovation, the Bill and Melinda Gates Foundation, the Manitoba Children’s Hospital Foundation, Prolacta Biosciences, Mitacs, CIFAR, the Garfield Weston Foundation, Health Data Research UK, and Canadian COVID Immunity Task Force. She regularly speaks at conferences and workshops on infant nutrition, some sponsored by Prolacta Biosciences, and has spoken at a conference sponsored by AstraZeneca. She has contributed without remuneration to online courses on breast milk and the infant microbiome produced by Microbiome Courses. She serves in a volunteer capacity for the International Society for Research on Human Milk and Lactation and as a member of the US Canada Joint Human Milk Initiative. She has consulted for DSM Nutritional Products and serves on the Malaika Vx Scientific Advisory Board.

The remaining authors declare that the research was conducted in the absence of any commercial or financial relationships that could be construed as a potential conflict of interest.

## Publisher’s note

All claims expressed in this article are solely those of the authors and do not necessarily represent those of their affiliated organizations, or those of the publisher, the editors and the reviewers. Any product that may be evaluated in this article, or claim that may be made by its manufacturer, is not guaranteed or endorsed by the publisher.

## Supplementary material

Supplementary Figure 1 Network representation of proteins in milk from the different mother-child allergy status groups from the CHILD Cohort Study. Networks are inferred from the adjacency matrices obtained with the Probabilistic Context Likelihood of Relatedness on Correlation (PCLRC) algorithm and are created with a circular layout. In the labeling of the groups, + indicates allergy and - indicates no allergy. It can be observed that there is less protein connectivity (fewer edges) in the group where mother and child are non-allergic when compared to the other groups.

Supplementary Table 1 Table with all identified proteins in human milk from the CHILD Cohort Study.
