# Corrigendum: The human milk proteome and allergy of mother and child: exploring associations with protein abundances and protein network connectivity

**DOI:** 10.3389/fimmu.2023.1276180

**Published:** 2023-09-18

**Authors:** Pieter M. Dekker, Meghan B. Azad, Sjef Boeren, Piushkumar J. Mandhane, Theo J. Moraes, Elinor Simons, Padmaja Subbarao, Stuart E. Turvey, Edoardo Saccenti, Kasper A. Hettinga

**Affiliations:** ^1^ Food Quality and Design Group, Wageningen University and Research, Wageningen, Netherlands; ^2^ Laboratory of Biochemistry, Wageningen University and Research, Wageningen, Netherlands; ^3^ Department of Pediatrics and Child Health, University of Manitoba, Winnipeg, MB, Canada; ^4^ Manitoba Interdisciplinary Lactation Centre (MILC), Children’s Hospital Research Institute of Manitoba, Winnipeg, MB, Canada; ^5^ Department of Pediatrics, University of Alberta, Edmonton, AB, Canada; ^6^ Division of Respiratory Medicine, Department of Pediatrics, Hospital for Sick Children, University of Toronto, Toronto, ON, Canada; ^7^ Department of Physiology, University of Toronto, Toronto, ON, Canada; ^8^ Department of Pediatrics, University of British Columbia, Vancouver, BC, Canada; ^9^ Laboratory of Systems and Synthetic Biology, Wageningen University and Research, Wageningen, Netherlands

**Keywords:** breastmilk, milk proteome, allergic disease, allergy development, immunology of human milk, differential network analysis, allergen, immunomodulatory

In the published article, there was an error. The data processing was carried out without setting the fixed propionamide modification for cysteines.

In the sample preparation carried out in our study, proteins were reduced and alkylated before tryptic digestion and analysis. This resulted in the modification of the cysteine residues within the sequence. After the nontargeted analysis, this modification should have been taken into account in the settings of the data processing in order to correctly identify all tryptic peptides. The error was that this modification was not set in the software, which resulted in false negative identification of peptides with cysteine residues. Consequently, the identification of proteins was only based on tryptic peptides with unmodified cysteines in their sequence, and proteins rich in cysteines were therefore also identified less.

After discovering this error, we reprocessed the raw data, setting the fixed modification in the data processing software. Although this reprocessing resulted in increased identification of cysteine-rich proteins and, consequently, in differences in numbers, tables, figures, and supplementary material, the discussion and conclusions of the article remain the same.

## Text Corrections

Corrections have been made to Materials and methods, *Statistical methods*, Missing data. This sentence previously stated:

In practice this resulted in a minimum of 66 and a median of 215 valid values.

The corrected sentence appears below:

In practice this resulted in a minimum of 49 and a median of 209 valid values.

Corrections have been made to Materials and methods, *Statistical methods*, Principal component analysis. This sentence previously stated:

For unsupervised data exploration, Principal Component Analysis (PCA) (40) was applied on the 300 × 647 data matrix (samples × proteins), using the FactoMineR package for R (41).

The corrected sentence appears below:

For unsupervised data exploration, Principal Component Analysis (PCA) (40) was applied on the 300 × 687 data matrix (samples × proteins), using the FactoMineR package for R (41).

Corrections have been made to Materials and methods, *Statistical methods*, Network inference and analysis - Covariance simultaneous component analysis (COVSCA). This sentence previously stated:

This fit was chosen as the best compromise between goodness of fit (68%) and the complexity of the COVSCA model (rank and number of the prototypical matrices).

The corrected sentence appears below:

This fit was chosen as the best compromise between goodness of fit (74%) and the complexity of the COVSCA model (rank and number of the prototypical matrices).

Corrections have been made to Results, paragraph one. This paragraph previously stated:

Proteomic analysis of all samples led to a total of 1629 identified proteins before filtering on missing values. After filtering these proteins on the requirement of being identified ≥ 25 times in at least one of the four mother-child allergy groups, 647 proteins remained for further data analysis. In this filtered dataset, the number of identified proteins per sample ranged between 256 and 586 (median = 458). The major milk proteins *α*-lactalbumin, albumin, lactoferrin, *β*-casein, and *α*_s1_-casein, were in all analyzed samples among the 15 most abundant proteins. A complete overview of the 647 identified proteins can be found in Supplementary Table 1.

The corrected paragraph appears below:

Proteomic analysis of all samples led to a total of 1690 identified proteins before filtering on missing values. After filtering these proteins on the requirement of being identified ≥ 25 times in at least one of the four mother-child allergy groups, 687 proteins remained for further data analysis. In this filtered dataset, the number of identified proteins per sample ranged between 242 and 636 (median = 480). The major milk proteins *α*-lactalbumin, albumin, lactoferrin, *β*-casein, and *α*_s1_-casein, were in all analyzed samples among the 15 most abundant proteins. A complete overview of the 687 identified proteins can be found in Supplementary Table 1.

Corrections have been made to Results, *Univariate analysis*. This section previously stated:

Differences in protein abundance between the different mother-child allergy groups were assessed with Kruskal-Wallis tests. After correction for multiple hypothesis testing, no significant differences were found among the four groups (**Table 1**). Kruskal-Wallis outcomes with uncorrected *p <* 0.05 were further assessed with Dunn’s *post-hoc* tests and subsequent correction for multiple testing, which resulted in 23 proteins that showed a difference between the groups with corrected *p <* 0.05 (**Table 1**). Most of these differences (*n* = 15) were found between the non-allergic group (M-C-) and the group where only the child ultimately developed an allergy (M-C+). Proteins that differed between these groups were primarily Ig chains (11 out of 15) and were mostly higher in abundance in the group where the mother was non-allergic and the child developed an allergy (**Figure 3**). Additionally, 4 of these Igs show also a higher abundance in milk from allergic mothers with children who did not develop an allergy. Further investigation of all identified Ig proteins showed that the mean abundance of these proteins is generally lower in the groups where mother, child or both are allergic, when compared to the non-allergic group (**Figure 4**). This effect is clearest in the comparison of the group where only the child developed an allergy with the group where both mother and child are nonallergic. Out of 81 Ig proteins, 75 have a mean abundance that is higher in the group where the child developed an allergy.

**Table 1:** Results of univariate analysis (Kruskal-Wallis) with subsequent *post-hoc* test (Dunn’s) for the comparison of protein abundance in milk from allergic (M+) and non-allergic (M-) mothers, with children who developed an allergy (C+) and did not develop an allergy (C-) in the CHILD Cohort Study.

					Comparison			
Leading protein	UniProt ID	Keyword	*p*-value*^a^*	Adjusted *p*-value*^a^*	Group 1	Group 2	Adjusted *p*-value*^b^*	Trend
Prosaposin	P07602	Lipid metabolism	0.046	0.915	M-/C-	M-/C+	0.035	⇓
N-acetylglucosamine-6-sulfatase	P15586	Hydrolase	0.040	0.915	M-/C-	M-/C+	0.040	⇓
IGL c830-light	A0A5C2FXC1	Immunoglobulin	0.031	0.813	M-/C-	M-/C+	0.022	⇓
V2-7 protein	A2MYD4	Immunoglobulin	0.014	0.813	M-/C-	M-/C+	0.017	⇓
IGL c1566-light	A0A5C2G1B3	Immunoglobulin	0.009	0.813	M-/C-	M-/C+	0.005	⇓
IGL c560-light	A0A5C2G943	Immunoglobulin	0.025	0.813	M-/C-	M-/C+	0.028	⇓
IGH + IGL c632-heavy	A0A5C2GC20	Immunoglobulin	0.004	0.644	M-/C-	M-/C+	0.002	⇓
IG c662-heavy	A0A5C2GE75	Immunoglobulin	0.002	0.644	M-/C-	M-/C+	0.002	⇓
IG c326-heavy	A0A5C2GF50	Immunoglobulin	0.012	0.813	M-/C-	M-/C+	0.013	⇓
IG c56-heavy	A0A5C2GL63	Immunoglobulin	0.001	0.644	M-/C-	M-/C+	0.006	⇓
IG c1713-heavy	A0A5C2GS26	Immunoglobulin	0.044	0.915	M-/C-	M-/C+	0.029	⇓
Lambda-chain	A2NUT2	Immunoglobulin	0.030	0.813	M-/C-	M-/C+	0.026	⇓
Immunoglobulin heavy	P01877	Immunoglobulin	0.003	0.644	M-/C-	M-/C+	0.002	⇓
Immunoglobulin alpha-2 heavy	P0DOX2	Immunoglobulin	0.017	0.813	M-/C-	M-/C+	0.014	⇓
Immunoglobulin kappa light	P0DOX7	Immunoglobulin	0.012	0.813	M-/C-	M-/C+	0.043	⇓
Immunoglobulin heavy	Q9NPP6	Immunoglobulin	0.048	0.915	M-/C-	M-/C+	0.036	⇓
IgG H chain	S6BGF5	Immunoglobulin	0.012	0.813	M-/C-	M-/C+	0.015	⇓
V2-7 protein	A2MYD4	Immunoglobulin	0.014	0.813	M-/C-	M+/C-	0.034	⇓
IGH c442-heavy	A0A5C2G767	Immunoglobulin	0.018	0.813	M-/C-	M+/C-	0.012	⇑
IGL c560-light	A0A5C2G943	Immunoglobulin	0.025	0.813	M-/C-	M+/C-	0.028	⇓
IG c56-heavy	A0A5C2GL63	Immunoglobulin	0.001	0.644	M-/C-	M+/C-	0.002	⇓
Trans-Golgi network integral membrane protein 2	O43493	Membrane protein	0.021	0.813	M-/C-	M+/C-	0.024	⇓
Synaptobrevin homolog YKT6	O15498	Transport	0.032	0.813	M-/C-	M+/C-	0.034	⇓
Dyslexia-associated protein KIAA0319-like protein	Q8IZA0	Membrane protein	0.005	0.644	M-/C-	M+/C-	0.003	⇑
RNA-binding region-containing protein 3	Q96LT9	RNA-binding	0.018	0.813	M-/C-	M+/C-	0.040	⇑
V2-7 protein	A2MYD4	Immunoglobulin	0.014	0.813	M-/C-	M+/C+	0.050	⇓
IGH + IGL c632-heavy	A0A5C2GC20	Immunoglobulin	0.004	0.644	M-/C-	M+/C+	0.040	⇓
IG c56-heavy	A0A5C2GL63	Immunoglobulin	0.001	0.644	M-/C-	M+/C+	0.031	⇓
Synaptobrevin homolog YKT6	O15498	Transport	0.032	0.813	M-/C-	M+/C+	0.034	⇓
Immunoglobulin heavy	P01877	Immunoglobulin	0.003	0.644	M-/C-	M+/C+	0.048	⇓
Immunoglobulin kappa light	P0DOX7	Immunoglobulin	0.012	0.813	M-/C-	M+/C+	0.043	⇓
IG c662-heavy	A0A5C2GE75	Immunoglobulin	0.002	0.644	M-/C+	M+/C-	0.018	⇑
Cathepsin C	B4DJQ8	Peptidase	0.026	0.813	M-/C+	M+/C-	0.019	⇑
Complement component C7	P10643	Complement protein	0.030	0.813	M-/C+	M+/C-	0.037	⇑
Inactive C-alpha-formylglycine-generating enzyme 2	Q8NBJ7	Metal-binding	0.019	0.813	M-/C+	M+/C+	0.048	⇓
Plectin	Q15149	Cell structure	0.034	0.836	M-/C+	M+/C+	0.046	⇓
Protein FAM3C	Q92520	Cytokine	0.007	0.790	M-/C+	M+/C+	0.004	⇑
Inactive C-alpha-formylglycine-generating enzyme 2	Q8NBJ7	Metal-binding	0.019	0.813	M+/C-	M+/C+	0.018	⇓
Matrilin-2	O00339	Matrilin	0.023	0.813	M+/C-	M+/C+	0.014	⇓
Trans-Golgi network integral membrane protein 2	O43493	Membrane protein	0.021	0.813	M+/C-	M+/C+	0.024	⇑
cDNA FLJ50830	B4DPR2	Albumin	0.022	0.813	M+/C-	M+/C+	0.034	⇓
Alpha-S1-casein	D6RF34	Transport	0.030	0.813	M+/C-	M+/C+	0.037	⇓
Immunoglobulin kappa light	P0DOX7	Immunoglobulin	0.012	0.813	M+/C-	M+/C+	0.043	⇓
Plectin	Q15149	Cell structure	0.034	0.836	M+/C-	M+/C+	0.048	⇓
RNA-binding region-containing protein 3	Q96LT9	RNA-binding	0.018	0.813	M+/C-	M+/C+	0.019	⇓

^a^from Kruskal-Wallis tests.

^b^ from Dunn’s post-hoc tests.

The trend indicates higher (⇑) or lower (⇓) abundance in the first group in the comparison. Listed are all proteins with uncorrected p-value < 0.05 (Kruskal-Wallis) and corrected p-value < 0.05 (Dunn’s), sorted by the mother-child allergy groups in the comparison.

**Figure 3:**
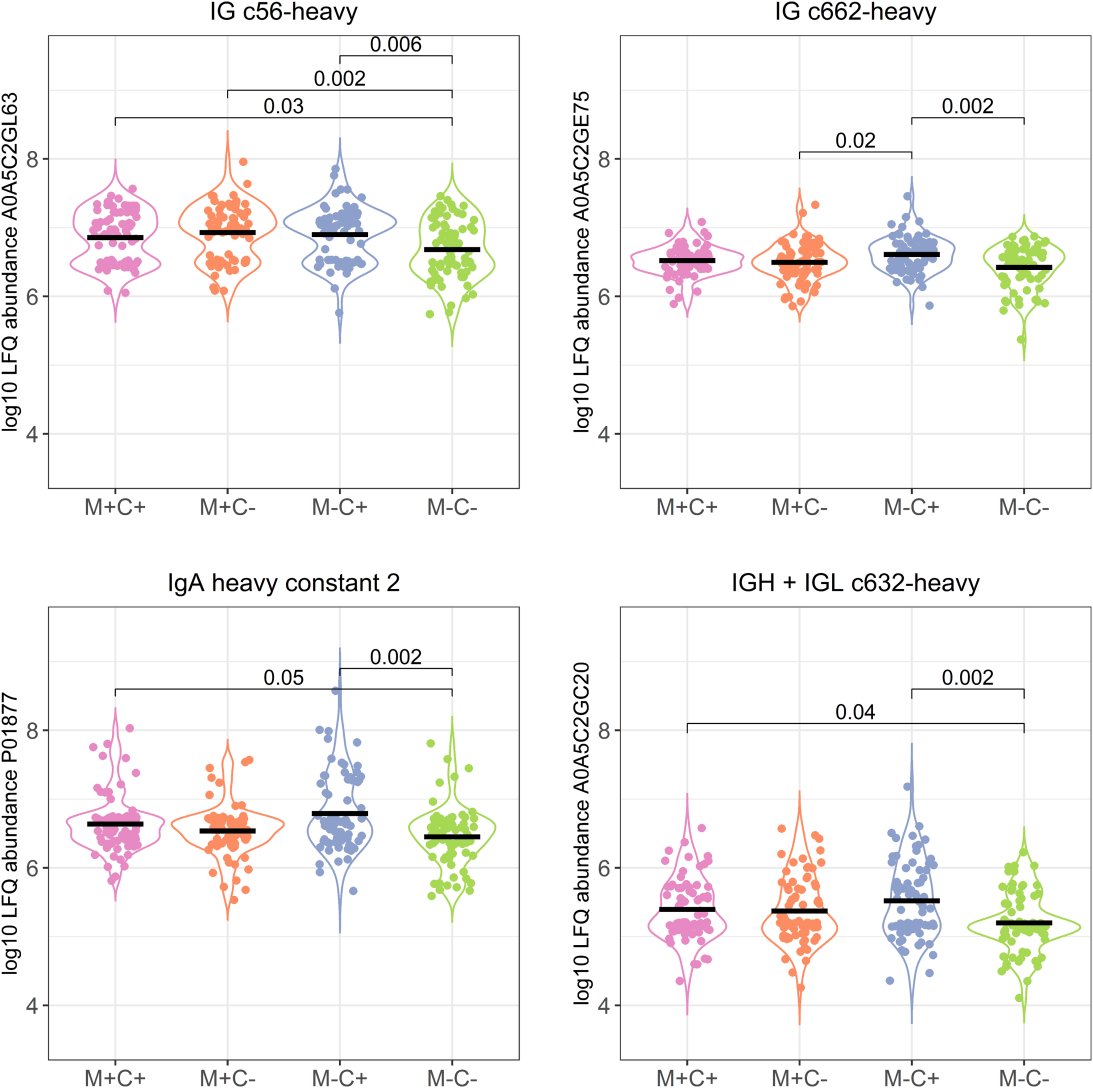
Violin plots visualizing the differences in abundance of the 4 most significantly different immunoglobulin (Ig) chains between the different allergy status groups from the CHILD Cohort Study. Differences between groups are indicated with *p*-values from Dunn’s *post-hoc* tests, and means of each group are shown with black, horizontal lines. In the labeling of the groups, M indicates mother, C indicates child, + indicates allergy, and - indicates no allergy.

**Figure 4:**
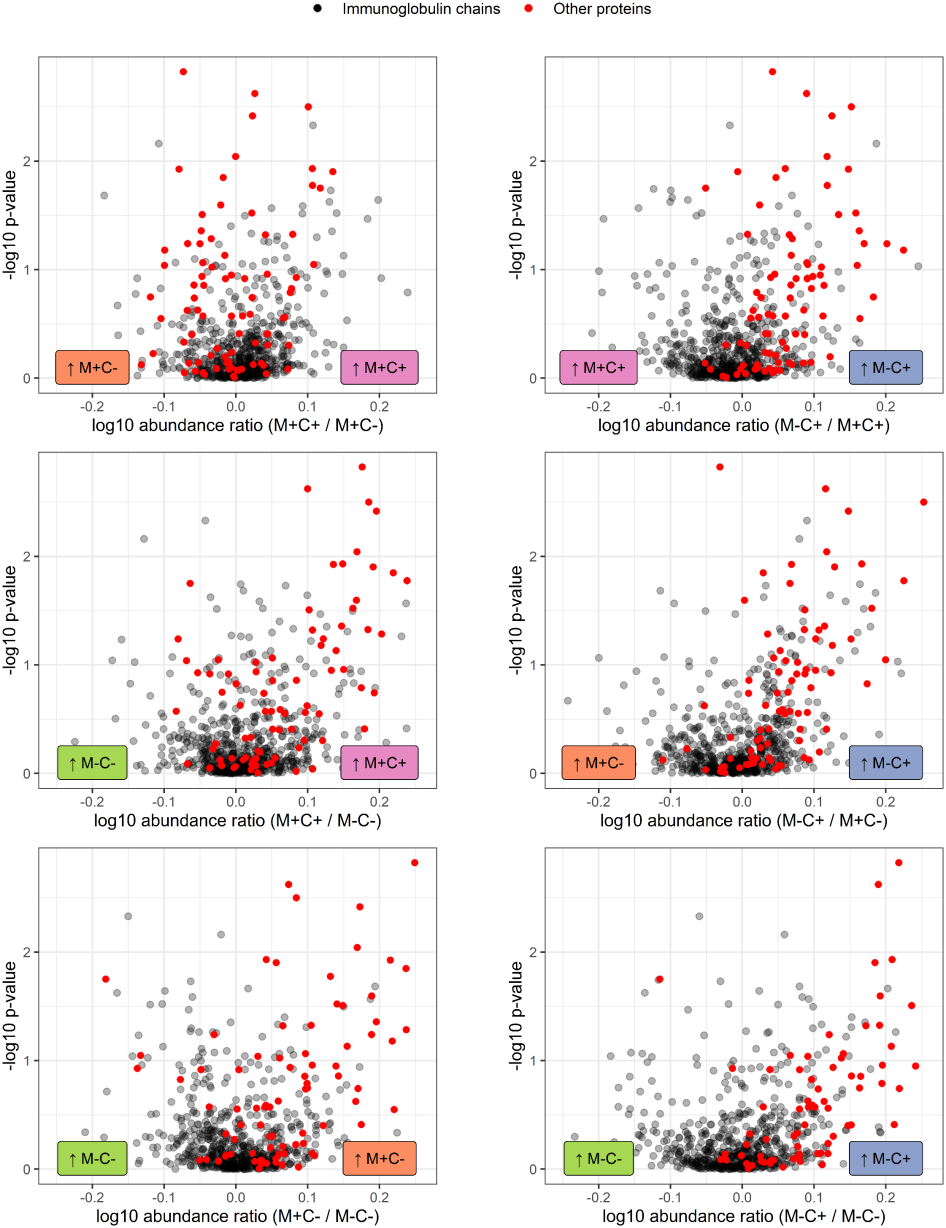
Volcano plots visualizing the trend in immunoglobulin abundances in milk from different mother-child allergy status groups from the CHILD Cohort Study. Each data point represents one protein, with on the x-axes the ratio of the means of the log10 transformed label-free quantification (LFQ). Immunoglobulin-related proteins are represented by red and other proteins with grey dots. Colored labels on left and right side of x = 0 indicate in which mother-child allergy status group the mean abundance of the respective proteins is higher. In the labeling of the groups, M indicates mother, C indicates child, + indicates allergy, and - indicates no allergy. A trend can be observed that most immunoglobulin-related proteins are higher in abundance in the group where the mother is non-allergic and the child ultimately develops an allergy.

The corrected section appears below:

Differences in protein abundance between the different mother-child allergy groups were assessed with Kruskal-Wallis tests. After correction for multiple hypothesis testing, no significant differences were found among the four groups (**Table 1**). Kruskal-Wallis outcomes with uncorrected *p <* 0.05 were further assessed with Dunn’s *post-hoc* tests and subsequent correction for multiple testing, which resulted in 30 proteins that showed a difference between the groups with corrected *p <* 0.05 (**Table 1**). Most of these differences (*n* = 17) were found between the non-allergic group (M-C-) and the group where only the child ultimately developed an allergy (M-C+). Proteins that differed between these groups were primarily Ig chains (15 out of 17) and were mostly higher in abundance in the group where the mother was non-allergic and the child developed an allergy (**Figure 3**). Additionally, 3 of these Igs show also a higher abundance in milk from allergic mothers with children who did not develop an allergy. Further investigation of all identified Ig proteins showed that the mean abundance of these proteins is generally lower in the groups where mother, child or both are allergic, when compared to the non-allergic group (**Figure 4**). This effect is clearest in the comparison of the group where only the child developed an allergy with the group where both mother and child are nonallergic. Out of 83 Ig proteins, 77 have a mean abundance that is higher in the group where the child developed an allergy.

Corrections have been made to Results, *Non-human proteins*, paragraph one. This sentence previously stated:

In the current study, several non-human proteins were identified (*n* = 9), including albumin from dog, horse, and cat, as well as bovine *α*_s1_-casein and BLG (**Table 2**).

**Table 2:** Identified non-human tryptic peptides in human milk samples from the CHILD Cohort Study (*n* = 150 allergic mothers and 150 non-allergic mothers).

Sequence	UniProt ID	Leading protein	Organism	Identified in *n* (%) samples from allergic mothers	Identified in *n* (%) samples from non-allergic mothers	Identification score^b^
KQTALVELLK	P49822	Albumin	Bos taurus (Bovine)	15 (10)	20 (13)	87.3
LVNELTEFAK	P02769	Albumin	Bos taurus (Bovine)	107 (71)	106 (71)	125.0
EKVNELSK	P02662	*α*_s1_-casein	Bos taurus (Bovine)	2 (1)	3 (2)	149.7
HIQKEDVPSER	P02662	*α*_s1_-casein	Bos taurus (Bovine)	7 (5)	2 (1)	93.6
FLDDDLTDDIMCVK	P00711	Alphalactalbumin	Bos taurus (Bovine)	12 (8)	14 (9)	164.6
FLDDDLTDDIMCVKK	P00711	Alphalactalbumin	Bos taurus (Bovine)	5 (3)	9 (6)	107.1
IDALNENK	P02754	*β*-lactoglobulin	Bos taurus (Bovine)	20 (13)	18 (12)	89.8
LISVDTEHSNIYLQNGPNR	F1N076	Ceruloplasmin	Bos taurus (Bovine)	40 (27)	37 (25)	203.6
MFTTAPDQVDKENEDFQESNK	F1N076	Ceruloplasmin	Bos taurus (Bovine)	2 (1)	2 (1)	88.0
VTISCSGGR	Q29RQ1	Complement component C7	Bos taurus (Bovine)	83 (55)	88 (59)	128.7
IVVDNKCK	Q3SYR8	Immunoglobulin J chain	Bos taurus (Bovine)	125 (83)	123 (82)	124.3
AQQHYPVSAGYTK	P11151	Lipoprotein lipase	Bos taurus (Bovine)	67 (45)	66 (44)	129.5
QEPDRNECFLAHK	P49822	Albumin	Canis lupus familiaris (Dog)	141 (94)	139 (93)	99.5
KCAADESAENCDKSLHTLFGDK	A0A8C4PRE0	Albumin	Equus caballus (Horse)	2 (1)	0 (0)	81.9
LVNEVTEFAKK	A0A8C4PRE0	Albumin	Equus caballus (Horse)	119 (79)	117 (78)	124.1
AEFAEISK	P49064	Albumin	Felis catus (Cat)	69 (46)	71 (47)	84.9
AFKAWSVAR	P49064	Albumin	Felis catus (Cat)	86 (57)	88 (59)	98.3
YICENQDSISTK	P49064	Albumin	Felis catus (Cat)	75 (50)	79 (53)	146.1

^a^Score from the MaxQuant output indicating the quality of the identification of the peptide. A higher score represents a better identification.

The corrected sentence appears below:

In the current study, several non-human proteins were identified (*n* = 11), including albumin from dog, horse, and cat, as well as bovine *α*_s1_-casein and BLG (**Table 2**).

Corrections have been made to Results, *Network analysis*, Network inference, paragraph 2. This paragraph previously stated:

To investigate this pattern further, proteins with differential connectivity *>* 50 were selected for further investigation (Supplementary Table 1). These proteins had the largest differences in connectivity among the four different groups and were selected for further functional analysis, to determine possible functional consequences of the differences between the networks. The selection resulted in 173, 171, and 153 proteins for the comparison of the group with non-allergic mother and child with respectively (*i*) allergic mother and non-allergic child, (*ii*) non-allergic mother and allergic child, and (*iii*) allergic mother and child groups. From these proteins, 95 proteins occurred in all three selections, showing a similarity in differential connectivity. Interestingly, GO overrepresentation analysis of these proteins showed a significant overrepresentation of proteins involved in translation initiation (*p* = 1.08×10^−15^). This overrepresentation is due to 24 ribosomal proteins and translation initiation factors (EIF3A, EIF4A1, EIF5A).

The corrected paragraph appears below:

To investigate this pattern further, proteins with differential connectivity *>* 50 were selected for further investigation (Supplementary Table 1). These proteins had the largest differences in connectivity among the four different groups and were selected for further functional analysis, to determine possible functional consequences of the differences between the networks. The selection resulted in 160, 168, and 144 proteins for the comparison of the group with non-allergic mother and child with respectively (*i*) allergic mother and non-allergic child, (*ii*) non-allergic mother and allergic child, and (*iii*) allergic mother and child groups. From these proteins, 79 proteins occurred in all three selections, showing a similarity in differential connectivity. Interestingly, GO overrepresentation analysis of these proteins showed a significant overrepresentation of proteins involved in translation (*p* = 9.13×10^−9^). This overrepresentation is due to 23 ribosomal proteins and translation elongation factor EEF1A1P5.

Corrections have been made to Results, *Network analysis*, Network modeling, paragraph 3. This paragraph previously stated:

The loadings for component 1 (see **Figure 8**) are overrepresented by proteins involved in gluconeogenesis (*p* = 0.0003), the synthesis of glucose. This component accounts for separation between the non-allergic group and the other three groups. The second component, which drives the separation of the groups on allergy status of the child, shows a significant overrepresentation of proteins involved in the positive regulation of DNA biosynthetic processes (*p* = 0.0013). This overrepresentation is mainly due to 5 members of the tailless complex polypeptide 1 ring complex (TRiC or CCT). In addition, several proteins involved in translation processes show differences in correlation patterns on this component.

The corrected paragraph appears below:

The loadings for component 1 (see **Figure 8**) are overrepresented by proteins involved in gluconeogenesis (*p* = 0.003), the synthesis of glucose. This component accounts for separation between the non-allergic group and the other three groups. The second component, which drives the separation of the groups on allergy status of the child, does not show a significant overrepresentation of gene ontology terms. However, among these proteins are 11 proteins involved in translation as well as 3 members of the tailless complex polypeptide 1 ring complex (TRiC or CCT).

Corrections have been made to Discussion, *Differences in immunoglobulin abundances between groups with different allergy statuses*, paragraph 6. This sentence previously stated:

Notably, soluble CD14, a protein in human milk that may be protective against the development of food allergies (56, 57), was not different between the allergy groups in our study (uncorrected *p* = 0.53).

The corrected sentence appears below:

Notably, soluble CD14, a protein in human milk that may be protective against the development of food allergies (56, 57), was not different between the allergy groups in our study (uncorrected *p* = 0.43).

## Error in Figure/Table

For the same reason as explained above, corrections have been made to **Tables 1**–**3**, and **Figures 3**–**8**.

**Figure 5:**
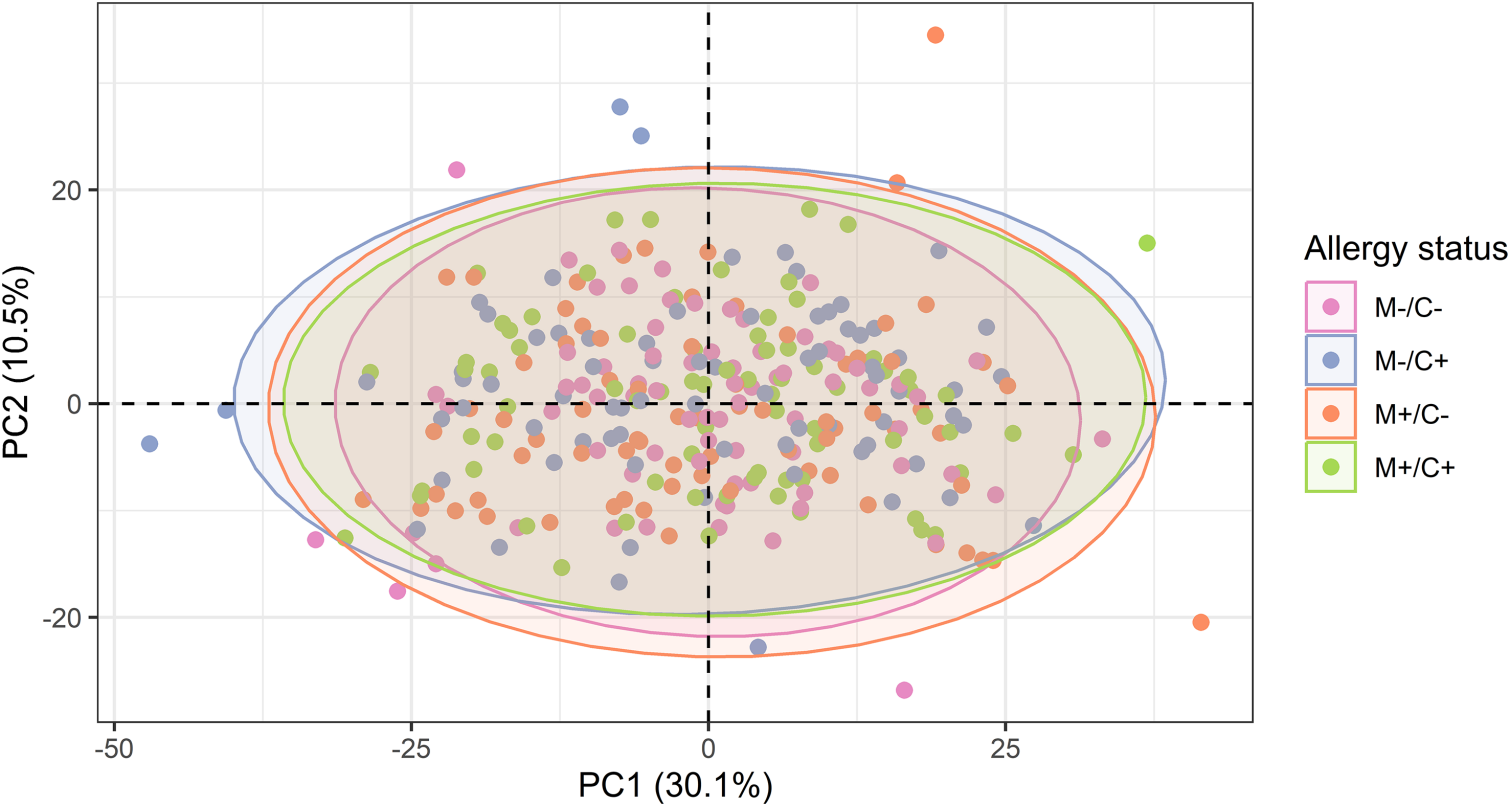
Scatter plot of principal component analysis (PCA) representing the human milk protein profile of mother-child dyads from the CHILD Cohort Study. Each point represents one dyad. No obvious differences can be observed among protein profiles of different mother-child allergy groups using this method.

**Figure 6:**
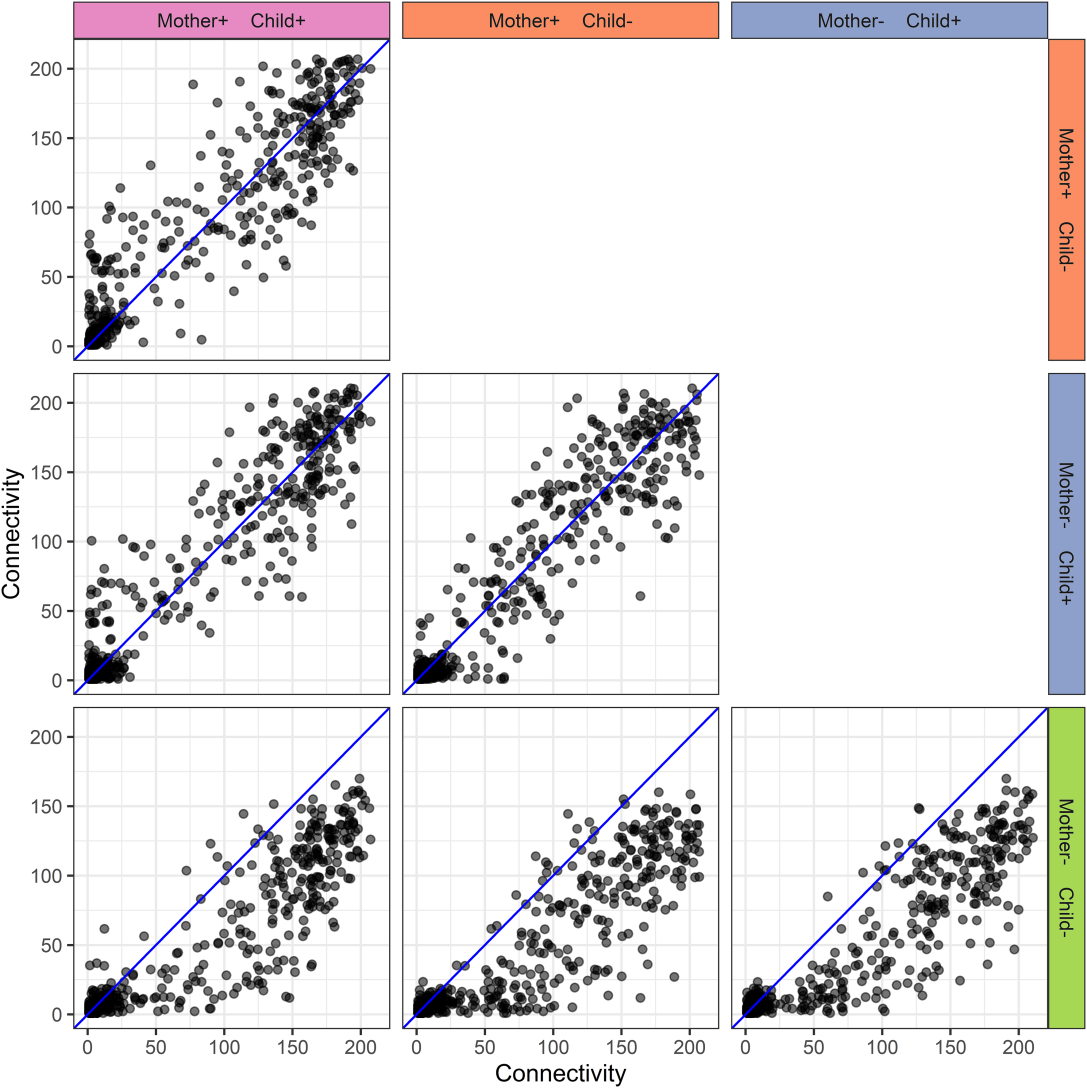
Human milk protein connectivity in the different mother-child allergy groups from the CHILD Cohort Study. Each subplot represents a pairwise comparison of protein connectivity in two mother-child allergy groups and each dot represents a single protein. Protein connectivity is obtained from the adjacency matrices build with the PCLRC algorithm and all groups are compared with one another in each subplot. In the labeling of the groups, + indicates allergy and - indicates no allergy. The group in which both mother and child are non-allergic shows a distinct connectivity pattern with an overall lower connectivity of the proteins.

**Figure 7:**
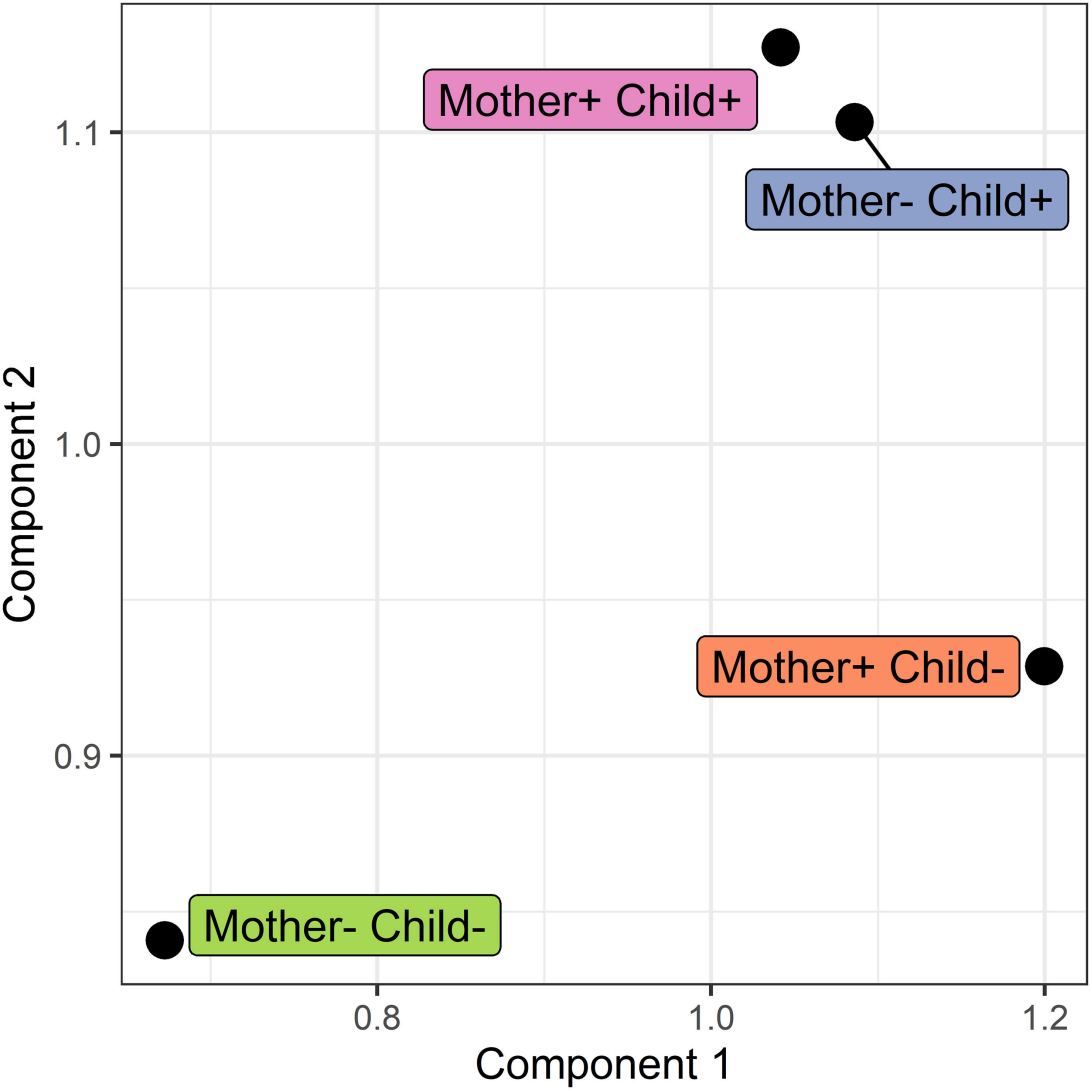
Score plot of the COVSCA model for the protein correlation network obtained using PCLRC of different groups based on maternal and child allergy status in the CHILD Cohort Study. Each point represents a protein-protein association network of one mother-child allergy group (+ indicates allergy, and- indicates no allergy). Protein importance for each component is shown in **Figure 8**. The groups with children who ultimately developed an allergy show similarities, whereas all the other groups show dissimilarities in correlation patterns.

**Figure 8:**
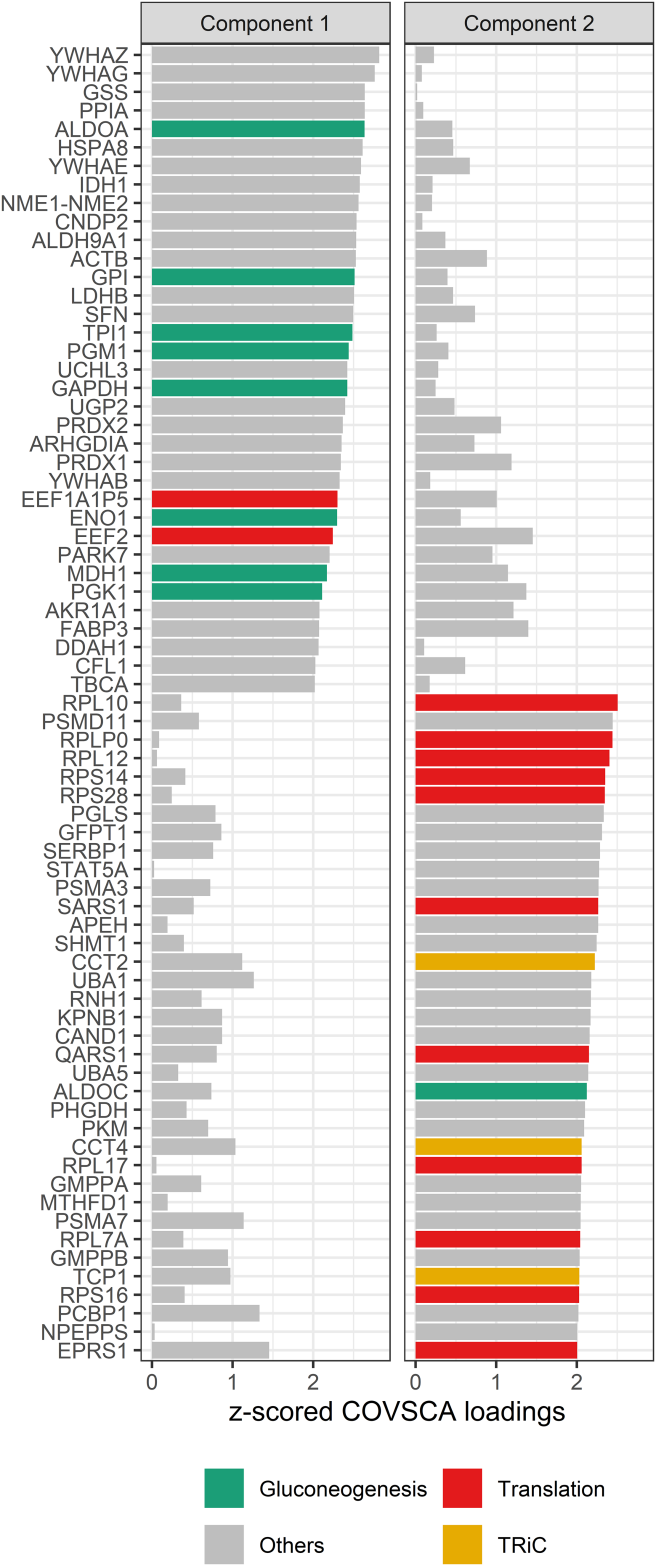
COVSCA loadings of the COVSCA model of different groups based on maternal and child allergy status in the CHILD Cohort Study. Loadings indicate the importance of each protein for the differences or similarities in correlation patterns observed in the COVSCA score plot (**Figure 7**). Proteins are labeled with gene IDs along the y-axis, and colors indicate shared gene ontology annotations. Among the proteins important for explaining the variability between the networks are proteins involved in gluconeogenesis, translation, and the tailless complex polypeptide 1 ring complex (TRiC).

**Table 3:** Outcome of Random Forest models on human milk proteins for the discrimination of groups with different allergy statuses from the CHILD Cohort Study.

Comparison					
Group 1	Group 2	Accuracy (%), (*p*-value)	Specificity (%), (*p*-value)	Sensitivity (%), (*p*-value)	AUROC, (*p*-value)
M+/C+	M+/C-	47.3 (0.57)	53.3 (0.18)	41.3 (0.84)	52.1 (0.74)
M+/C+	M-/C+	42.0 (0.87)	45.3 (0.62)	38.7 (0.93)	61.0 (0.12)
M+/C+	M-/C-	51.3 (0.28)	48.0 (0.46)	54.7 (0.14)	52.4 (0.72)
M+/C-	M-/C+	50.0 (0.37)	52.0 (0.24)	48.0 (0.50)	50.7 (0.91)
M+/C-	M-/C-	46.7 (0.62)	44.0 (0.71)	49.3 (0.42)	51.5 (0.83)
M-/C+	M-/C-	60.0 (0.01)	65.3 (0.00)	54.7 (0.18)	64.3 (0.04)

Comparisons of the groups are labelled according to allergy status, with allergic (M+) and non-allergic (M-) mothers, and allergic (C+) and non-allergic (C-) children.

The corrected Tables and Figures appear below.

## Incorrect Supplementary Material

For the same reason as explained above, corrections have been made to Supplementary Figure 1 and Table 1.

The correct Supplementary Material has been added to the original article.

The authors apologize for these errors and state that this does not change the scientific conclusions of the article in any way.

