# Supplementary figures and images for: The human milk proteome and allergy of mother and child: Exploring associations with protein abundances and protein network connectivity
Source: Front Immunol. 2022 Oct 13;13:977470. doi: 10.3389/fimmu.2022.977470 (PMC9613325; doi:10.3389/fimmu.2022.977470)

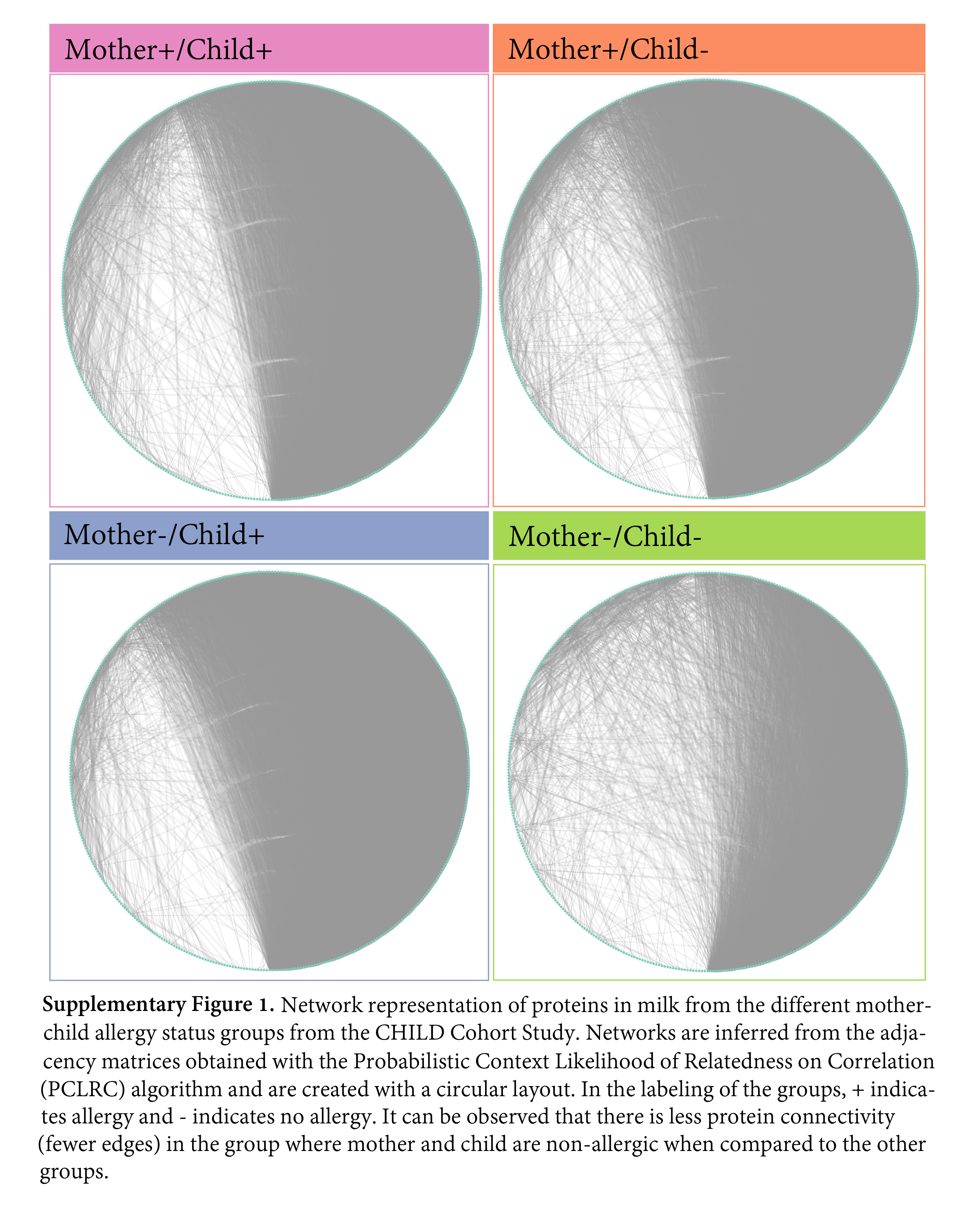

Supplement: Supplementary Figure 1 — Network representation of proteins in milk from the different mother-child allergy status groups from the CHILD Cohort Study. Networks are inferred from the adjacency matrices obtained with the Probabilistic Context Likelihood of Relatedness on Correlation (PCLRC) algorithm and are created with a circular layout. In the labeling of the groups, + indicates allergy and - indicates no allergy. It can be observed that there is less protein connectivity (fewer edges) in the group where mother and child are non-allergic when compared to the other groups. [file Image_1.png]
